# Acclimation of Photosynthesis to Changes in the Environment Results in Decreases of Oxidative Stress in *Arabidopsis thaliana*

**DOI:** 10.3389/fpls.2021.683986

**Published:** 2021-09-23

**Authors:** Mohd Fauzihan Karim, Giles N. Johnson

**Affiliations:** Department of Earth and Environmental Sciences, Michael Smith Building, University of Manchester, Manchester, United Kingdom

**Keywords:** light stress, oxidative stress, photosynthetic acclimation, antioxidants, reactive oxygen species

## Abstract

The dynamic acclimation of photosynthesis plays an important role in increasing the fitness of a plant under variable light environments. Since acclimation is partially mediated by a glucose-6-phosphate/phosphate translocator 2 (GPT2), this study examined whether plants lacking GPT2, which consequently have defective acclimation to increases in light, are more susceptible to oxidative stress. To understand this mechanism, we used the model plant *Arabidopsis thaliana* [accession Wassilewskija-4 (Ws-4)] and compared it with mutants lacking GPT2. The plants were then grown at low light (LL) at 100 μmol m^−2^ s^−1^ for 7 weeks. For the acclimation experiments, a set of plants from LL was transferred to 400 μmol m^−2^ s^−1^ conditions for 7 days. Biochemical and physiological analyses showed that the *gpt2* mutant plants had significantly greater activity for ascorbate peroxidase (APX), guiacol peroxidase (GPOX), and superoxide dismutase (SOD). Furthermore, the mutant plants had significantly lower maximum quantum yields of photosynthesis (Fv/Fm). A microarray analysis also showed that *gpt2* plants exhibited a greater induction of stress-related genes relative to wild-type (WT) plants. We then concluded that photosynthetic acclimation to a higher intensity of light protects plants against oxidative stress.

## Highlights

- The plants that are defective in their ability to acclimate their photosynthetic capacity to an increase in growth irradiance experience more oxidative stress.

## Introduction

The photosynthetic apparatus of plants is in the front-line of their response to the environment. Light varies across the day by up to three orders of magnitude. For instance, the light incident on a leaf fluctuates on a second timescale with changes in cloud cover or leaf movement. This fluctuation may also happen over hours through a diurnal cycle and over timescales of days to months with changing weather patterns through the year (Vialet-Chabrand et al., 2017; Slattery et al., 2018). Against this background, plants must seek to optimise their growth and fitness by absorbing light and capturing the energy through photosynthesis.

In the leaf, light is absorbed by chlorophyll molecules bound to light-harvesting complexes and core antenna proteins in the thylakoid membrane. In thus process, energy is transferred to reaction centres where charge separation results in a conversion to chemical energy, thus driving electron transport. This, in turn, drives the formation of the first stable products of photosynthesis, namely, NADPH and ATP, which are used in the Benson-Calvin cycle to fix atmospheric carbon dioxide (CO_2_) (Arnon, 1971; Cheng and Fleming, 2009).

Under optimal conditions, the absorption of light matches the capture of energy through CO_2_ fixation and other metabolic processes. However, if the light absorption exceeds metabolic capacity, the excess energy can instead drive alternative and harmful reactions that involve the formation of reactive oxygen species (ROS) (Tripathy and Oelmuller, 2012). Two distinct pathways give rise to ROS. The first occurs in photosystem II (PSII), wherein charge recombination reactions lead to the formation of triplet chlorophyll molecules. These interact with molecular oxygen (O) to form reactive singlet excited oxygen (^1^${\text{O}}_{2}^{*}$) (Cardona et al., 2012; Fischer et al., 2013; Pospíšil, 2016). The second pathway occurs when the reduced carriers in the electron transport chain, notably reduced iron sulphur clusters on the stromal side of photosystem I (PSI), can reduce O, with this reduction then generating superoxide radicals, ${\text{O}}_{2}^{-}$, in a process called the Mehler reaction (Mehler, 1951; Takagi et al., 2016; Choudhury et al., 2017). Even under optimal conditions, a certain proportion of light energy will go to generate ROS. However, when metabolic capacity is saturated, the rate of ROS production increases, which can lead to extensive damage to cell structures and even death in extreme cases.

Plants have evolved an array of different antioxidant systems to minimise damage from ROS (Jajic et al., 2015). For instance, carotenoids bound to light-harvesting and reaction centre proteins quench triplet chlorophylls and ^1^${\text{O}}_{2}^{*}$ (Pospíšil, 2012; Kvíčalová et al., 2016). Systems based around enzymes and soluble antioxidants are also important in detoxifying superoxide and derived radicals. Notably, superoxide is converted to hydrogen peroxide by superoxide dismutase (SOD). A range of peroxidases, including ascorbate peroxidase (APX) and glutathione peroxidase (GPX), also convert hydrogen peroxide into water (Myouga et al., 2008; Anjum et al., 2014; Yang et al., 2019).

In addition to detoxifying ROS, plants also possess mechanisms to minimise their formation. Under high light (HL) conditions, a cyclic electron flow results in the generation of a large ΔpH gradient across the thylakoid membrane (Johnson, 2011; Shikanai and Yamamoto, 2017). This, in turn, triggers a process called high-energy-state quenching, which is a form of non-photochemical quenching (NPQ) that dissipates excess energy as heat and reduces ^1^${\text{O}}_{2}^{*}$ generation (Björkman and Demmig-Adams, 1995; Ruban, 2016). At the same time, the downregulation of the cytochrome b_6_f complex prevents the overreduction of PSI and so limits ${\text{O}}_{2}^{-\cdot }$ formation (Laisk et al., 2005; Johnson, 2011; Chaux et al., 2015). These processes are induced and inactivated in response to changing conditions and reduce overall photosynthetic efficiency as a result of fluctuating conditions. The importance of these forms of regulation is emphasised by the studies on mutants that show that plants lacking such mechanisms have either reduced fitness or can even be non-viable in fluctuating environments (Suorsa et al., 2012; Gerotto et al., 2016; Nikkanen et al., 2018; Takagi and Miyake, 2018).

Over longer time scales, such as during days to weeks, plants may adjust the composition of their photosynthetic apparatuses through a process of photosynthetic acclimation (Walters, 2005; Herrmann et al., 2019). For instance, when a plant grown in one set of conditions is transferred to another, it will typically change its maximum capacity for photosynthesis (Yin and Johnson, 2000; Athanasiou et al., 2010; Suorsa et al., 2012; Spetea et al., 2014). This change results from a change in the relative investment of the plant in the different protein complexes involved. For example, a plant transferred to higher light might decrease its investment in light-harvesting complexes, whilst increasing its investment in reaction centres. This would result in an increase in the chlorophyll *a*:*b* ratio without changing leaf chlorophyll content (Kitajima and Hogan, 2003; Bailey et al., 2004). At the same time, increased investments in electron transfer proteins and Benson-Calvin cycle enzymes increase the capacity of metabolism. Thus, the overall effect of these changes is the increase in the ability of a plant to use the higher irradiance absorbed by its leaves (Miller et al., 2017).

Previously, we identified a mutant of *Arabidopsis thaliana* that lacks a chloroplast glucose-6-phosphate/phosphate translocator 2 (GPT2), making it deficient in its ability to increase photosynthetic capacity when transferred from low light (LL) to HL (Athanasiou et al., 2010). Glucose-6-phosphate/phosphate translocator 2 is one of the two genes encoding GPT2 in *A. thaliana*. The other gene, GPT1, is essential for embryo development (Andriotis et al., 2010; Zhang et al., 2020). Meanwhile, GPT2 has been shown to be induced under conditions where sugar concentrations in the leaf were increased (Lloyd and Zakhleniuk, 2004; Dyson et al., 2014). A recent work has also shown that this upregulation is linked to the expression of the redox responsive transcription factor 1 (RRTF1) (Weise et al., 2019). Furthermore, the photosynthetic acclimation response to an increase in light, *dynamic* acclimation, involves a change in the photosynthetic capacity of pre-developed tissues. This response is distinct from *developmental* acclimation, which occurs when leaves develop under a particular set of conditions. As a result, when *gpt2* mutant plants develop at HL, they produce leaves with the same photosynthetic capacity as their corresponding wild-type (WT) counterparts, such as Wassilewskija-4 (Ws-4). However, when the mature leaves of these plants are exposed to HL, they do not acclimate substantially (Athanasiou et al., 2010). Mutant plants also showed lower rates of steady-state photosynthesis at HL conditions compared with acclimated WT plants (Dyson et al., 2015). Supporting this, a detailed study also found that metabolic acclimation to HL was influenced by the active role of GPT2 in plants (Dyson et al., 2015). Meanwhile, mutants lacking the GPT2 protein were observed to have lower germination rates and slow seedling growth compared with WT plants (Dyson et al., 2014). In summary, the failure to acclimate to HL has been shown to result in plants being maintained in a state where their photosynthetic apparatus is more light-saturated. Therefore, in this study, we demonstrated that the failure to acclimate also resulted in additional costs to the plant, specifically in an increase in oxidative stress, which further resulted in increased investments in antioxidant systems. This showed that photosynthetic acclimation is a central process in determining plant fitness and productivity, which operate upstream of protective and repair mechanisms. Thus, we suggested that photosynthetic acclimation is an under-considered target for improving crop performance.

## Materials and Methods

### Plant Material and Growth Conditions

The seeds of *A. thaliana* accession Ws-4 and T-DNA insertion mutant *gpt2* (FLAG_326E03; INRA, Versailles, France) were grown on 8-h days at an irradiance of 100 μmol.m^−2^.s^−1^ LL and a temperature of 20°C during the day and 16°C at night in 7.62-cm pots filled with peat-based multipurpose compost. Short-day conditions were used to generate plants with large leaves without initiating flowering. Growth light was also provided by warm white LEDs (LEDengin, San Jose, CA, USA) (colour temperature 3,000–3,200 K). The plants were grown for 8 weeks prior to the beginning of the experiments. Prior to these experimental treatments, however, the youngest fully expanded leaves were identified to avoid using the leaves that developed during HL exposure. These leaves were then selected for all the measurements in this study. For the acclimation experiments, randomly selected plants were transferred to a shelf in the same growth cabinet that was set to a light intensity of 400 μmol.m^−2^.s^−1^ HL_._ The plants were then maintained at this irradiance for up to 7 days.

### Gas Exchange Analysis

Maximum photosynthetic activity was measured on fully expanded leaves using an LI-6400XT portable photosynthesis system (LI-COR Biosciences, Lincoln, Nebraska USA). The maximum photosynthetic rate (P_max_) was measured under the conditions of 2,000 μl L^−1^ CO_2_ and 1,500 μmol.m^−2^.s^−1^. The leaves were then illuminated for 20 min before reading to allow for the achievement of a steady rate of photosynthesis.

### Transcriptomic Data Collection

Samples were taken after 4 h of illumination on Day 7 of the treatment. For each condition, a single mature leaf was detached from each of the three plants and immediately flash-frozen in liquid nitrogen (LN). Total RNA was extracted using an RNeasy Plant Mini kit (Qiagen, Crawley, UK). At least 200 ng of RNA were then biotinylated and hybridised to an Arabidopsis ATH1-121501 (Affymetrix, Santa Clara, CA, USA) oligonucleotide array (according to the instructions of the manufacturers). An Agilent GeneArray scanner 3000 7G using Affymetrix GeneChip^®^ Operating Software v1.4 was also used to read the arrays before quality control was performed using a dChip to cheque for any outliers (Li and Wong, 2001) (Aglient, Santa Clara, CA, USA; Affymetrix, Santa Clara, CA, USA). An analysis on the gene expression and normalisation was then carried out following a study by Bolstad et al. (2003). Transcriptomic data collected in this study are available from ArrayExpress (https://www.ebi.ac.uk/arrayexpress/experiments/E-MTAB-10282/).

### Data Analysis and Software

Transcriptomic data were analysed using different approaches to discover the behaviour of these data in response to HL. To discover the biological enrichment of the data uploaded, they were uploaded into the online tool Database for Annotation, Visualisation, and Integrated Discovery (DAVID) (https://david.ncifcrf.gov; Huang et al., 2008, 2009). Gene ontology (GO) was also performed using a web-based tool and database for GO analysis (http://bioinfo.cau.edu.cn/agriGO/analysis.php; Du et al., 2010).

The differential gene expression in the microarray was analysed with a modified *t*-test on logarithmically scaled data using Cyber-T (Baldi and Long, 2001). The filtering of the differentially expressed genes (DEGs) was performed using the criteria of *p* < 0.05 and *M*-fold ≥2 in one condition. The gene annotation used was derived from The Arabidopsis Information Resource (TAIR) (Stanford, CA, USA; http://www.arabidopsis.org/). The Affymetrix chip analysis was performed at the Microarray Facility of the University of Manchester (Manchester, UK).

### Chlorophyll Determination

The total chlorophyll content was extracted and measured according to a study by Porra et al. (1989). A leaf was taken and scanned with a flatbed scanner. The area was then estimated using the ImageJ software (https://imagej.nih.gov/ij/), Canon LIDE120 flatbed scanner (Canon, Tokyo, Japan). Afterwards, the leaves were ground using a pestle and mortar in 5 ml of 80% v/v acetone. The extracts were centrifuged (13,000 g) for 5 min and the supernatant was collected. The absorbance was then measured using an Ocean Optics USB4000 spectrophotometer (Ocean Optics, Dunedin, FL, USA).

### Chlorophyll Fluorescence Analysis

The emission of chlorophyll fluorescence was measured using a PAM-101 chlorophyll fluorometer (WALZ, Heinz Walz, Effeltrich, Germany) and calculated according to a study by Maxwell and Johnson (2000). A leaf from each plant was placed in the dark for 30 min prior to measurement. Then, a 1-s flash of light (6,800–7,500 μmol m^−2^ s^−1^) was given to saturate all the PSII reaction centres. Maximum quantum yield was measured as the ratio F_v_/F_m_ = (F_m_-F_o_)/F_m_.

### Enzyme Activity Assays

Four to five mature fully expanded leaves at ~0.3–0.5 g fresh weight were detached from each plant and immediately flash-frozen in liquid dinitrogen (LN_2_). The samples were collected under growth conditions at the end of the photoperiod. Each sample was then homogenised in 50 mM of a potassium phosphate buffer (pH 7), 1 mM of ethylenediaminetetraacetic acid (EDTA), 1 mM of ascorbic acid (AsA), and 1% w/v polyvinylpyrrolidone (PVP 40) before centrifugation at 15,000 g for 10 min. The supernatants were collected, frozen in LN_2_, and stored at −80°C for total ascorbate peroxidase (APX), guiacol peroxidase (GPOX), and superoxide dismutase (SOD) assays (Jiang and Zhang, 2001).

The total APX activities were recorded by observing absorbance changes at 290 nm using an extinction coefficient of 2.8 mM^−1^ cm^−1^ in a 2-ml reaction mixture containing 50 mM of a potassium phosphate buffer (pH 7), 0.1 mM of hydrogen peroxide (H_2_0_2_) 30%, 0.5 mM of AsA, and 40 μl of enzyme extract (Nakano and Asada, 1981).

The total GPOX activities were determined according to method of a study by Nakano and Asada (1981). A 2-ml reaction mixture containing 50 mM of a potassium phosphate buffer (pH 7), 10 mM of guaiacol, 0.1 mM of EDTA [prepared in 50 mM of a potassium phosphate buffer (pH 7)], 0.1 mM of H_2_0_2_ 30%, and 40 μl of an enzyme extract. The absorbance was recorded at 470 nm to follow the formation of tetraguaiacol (extinction coefficient 26.6 mM^−1^ cm^−1^).

The total SOD activities were measured by observing the formation of a blue colour, which indicates the photochemical reduction of nitro blue tetrazolium (NBT), in the assay. Intense blue light (>5,000 μmol m^−2^ s^−1^) was used to initiate the reaction in a 3-ml assay containing 50 mM of a potassium phosphate buffer (pH 7.8), 0.1 mM of EDTA, 75 μM of NBT, 13 mM of methionine, 20 μl of a leaf extract, and 2 μM of riboflavin. Furthermore, one unit of SOD activity was defined as the amount of enzymes required to inhibit a 50% reduction of NBT at 560 nm. The illumination was applied for 5 min for each sample (Giannopolitis and Ries, 1977).

### Lipid Peroxidation

The effect of HL on lipid peroxidation was examined through measurements of malondialdehyde (MDA) (Hodges et al., 1999). Two to three leaves at ~0.2–0.4 g fresh weight were sampled at the end of the Day 7 photoperiod and immediately flash-frozen in LN_2_. The plant tissue samples were homogenised in 3 ml of 0.1% (w/v) trichloroacetic acid (TCA) using a mortar and pestle on ice, followed by centrifugation at 10,000 g for 10 min. A 750-μl aliquot was then taken and mixed vigorously with same amount of either (a) a +thiobarbituric acid (TBA) solution containing 20% (w/v) TCA and 0.5% (w/v) TBA, or (b) –TBA containing only 20% (w/v) TCA in a 2-ml capped microcentrifuge tube. Afterwards, the samples were heated to 95°C for 25 min in a temperature-controlled water bath and then subsequently allowed to cool at room temperature prior to centrifugation at 10,000 g for 10 min. The absorbances were recorded using an Ocean Optics USB4000 spectrophotometer at 440, 532, and 600 nm.

### Antioxidant Assays

The determination of total ascorbate and dehydroascorbate was based on a method by Kampfenkel et al. (1995). Fully develop leaves were harvested and flash-frozen in liquid N_2_. Extraction was carried out immediately after harvest to minimise the oxidation from AsA to dehydroascorbate (DHA). Frozen leaves (0.2–0.4 g) were ground with 1.5 ml of 6% (w/v) TCA to a fine powder in a precooled mortar and pestle (with liquid N_2_). Homogenisation was continued until the mixture thawed and then left to stand for 5 min on ice. After transfer into a new 2-ml microcentrifuge tube, the homogenate was then centrifuged at 15,600 g for 5 min (4°C). The assays of AsA and DHA were carried out immediately at 525 nm.

Meanwhile, the measurement of relative anthocyanin content was described by in a study by Neff and Chory (1998). A fully developed leaf was homogenised in 5 ml of 60% methanol containing 1% hydrochloric acid: 40% Milli-Q water and centrifuged at 16,300 g for 5 min. A 700-μl aliquot was vigorously vortexed with the same amount of chloroform to separate chlorophylls from anthocyanins. Total relative anthocyanins were measured by subtracting A_657_ from A_530_ using an Ocean Optics USB4000 spectrophotometer.

### Statistical Analyses

Where appropriate, data were analysed using the GraphPad Prism software package (Graphpad software San Diego, CA, USA) (http://www.graphpad.com). The significance of different data was assessed using two-way ANOVAs followed by Tukey *post-hoc* tests. Significantly different values were represented on graphs using different letters.

## Results

### Plants Lacking GPT2 Are Deficient in Acclimation to HL and Experience Greater Photoinhibition and Oxidative Stress

**Figure 1:**
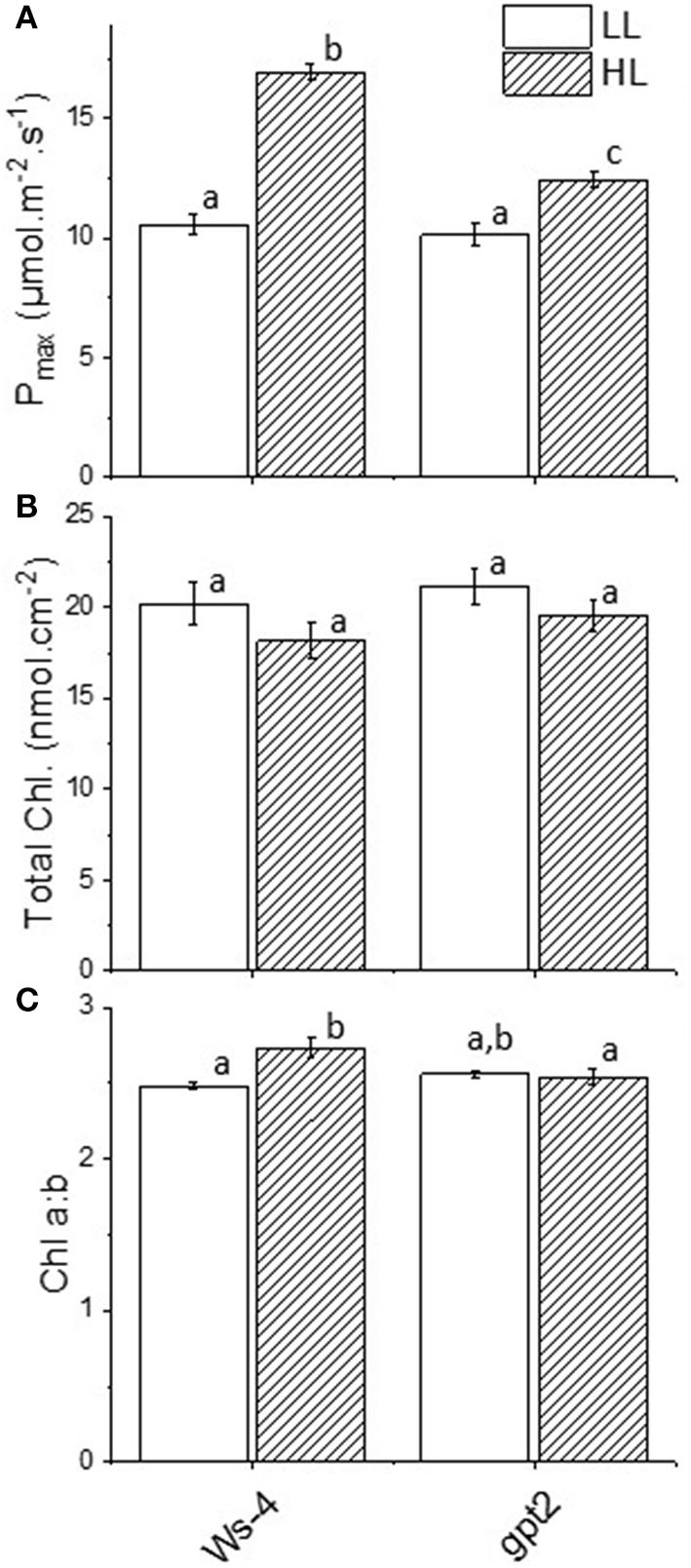
Acclimation responses in Wassilewskija-4 (Ws-4) wild type and glucose-6-phosphate/phosphate translocator 2 (*gpt2*) mutant plants. Plants were grown for 7 weeks at an irradiance of 100 μmol m^−2^ s^−1^ before either remaining at that irradiance (low light; LL) or being transferred to 400-μmol m^−2^ s^−1^ (high light; HL) conditions for 7 more days. **(A)** Photosynthetic capacity (P_max_) measured as the rate of photosynthesis in 2,000 μl L^−1^ CO_2_ and 1,500 μmol m^−2^ s^−1^ light. **(B)** Total chlorophyll content. **(C)** Chlorophyll *a*:*b*. Significance of the treatments were indicated with different lowercase letters, at *p* ≤ 0.05 (ANOVA/Tukey, *n* =4).

The plants of the Arabidopsis accession Ws-4 and mutant *gpt2* were grown to maturity for 8 weeks at an irradiance of 100 μmol.m^−2^.s^−1^ and then transferred to 400 μmol.m^−2^.s^−1^ for 7 days. Wassilewskija-4 plants showed significant increases in their maximum capacities for photosynthesis (P_max_), from around 10 to nearly 17 μmol CO_2_.m^−2^.s^−1^ (Figure 1A). At the same time, total leaf chlorophyll content did not change significantly (Figure 1B), but there was a change in the chlorophyll *a:b* ratio that increased in response to increased light (Figure 1C). In the *gpt2* mutant, there was a significant increase in P_max_ following acclimation to HL. However, this increase was significantly smaller than that seen in Ws-4 (Figure 1A). The chlorophyll *a:b* ratio did not change in response to high light in these plants (Figure 1C). These results are consistent with the previous observations of the same plant lines that were grown using different light conditions (Athanasiou et al., 2010; Dyson et al., 2015).

**Figure 2:**
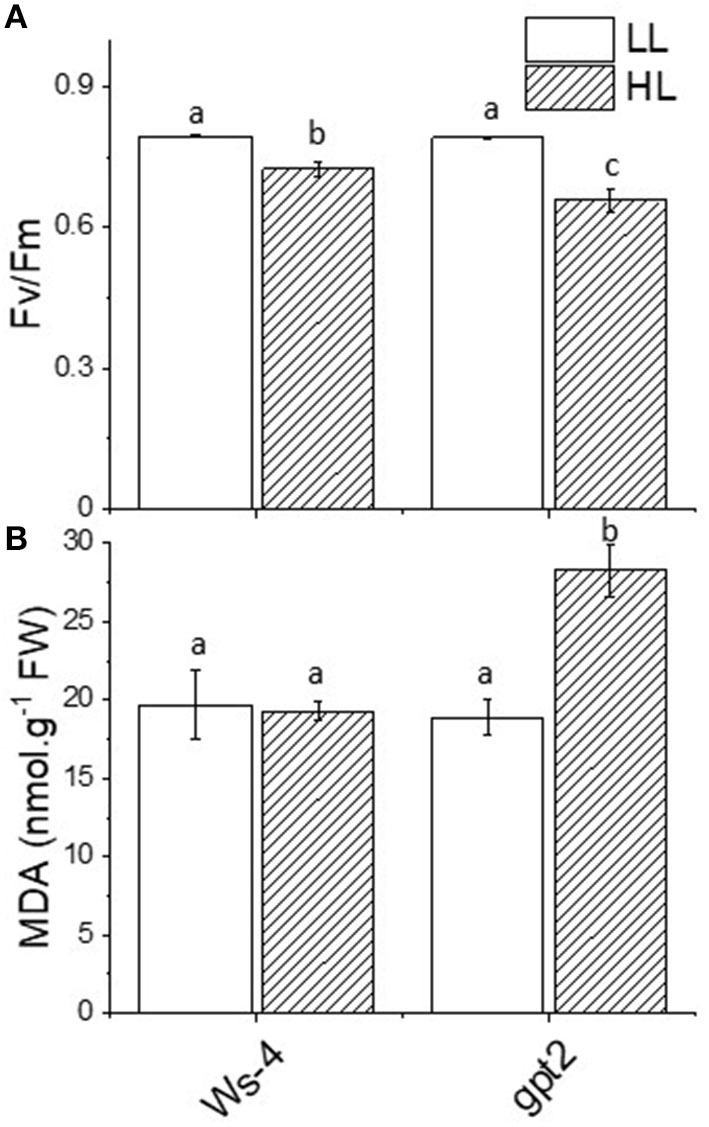
Stress responses in Ws-4 wild type and *gpt2* mutant plants. Plants were grown for 7 weeks at an irradiance of 100 μmol m^−2^ s^−1^ before either remaining at that irradiance (LL) or being transferred to 400-μmol m^−2^ s^−1^ (HL) conditions for 7 more days. **(A)** Maximum quantum yield of Photosystem II (F_v_/F_m_). Prior to the measurement, the plants were removed from the growth conditions and dark-adapted for 30 min. **(B)** Production of the malondialdehyde equivalent level (nmol g^−1^FW). Samples were harvested directly from growth conditions at mid-day on Day 7 of the treatment and flash-frozen in liquid N_2_. A two-way ANOVA followed by a Tukey's *post-hoc* test were used to test the significance between means. Significantly different values are indicated by different lowercase letters at *p* ≤ 0.05. Factorial analysis shows that both light and genotype factors are significant at *p* ≤ 0.05.

The measurements of chlorophyll fluorescence were undertaken to assess the impact of increased irradiance on photosynthetic performance. The ratio F_v_/F_m_ is widely used as an indicator of photoinhibition- and light-induced damage to the PSII reaction centre. In the Ws-4 WT, the dark-adapted F_v_/F_m_ underwent a small but significant decrease in response to 7 days of HL (Figure 2A). In the *gpt2* mutants, the decrease in F_v_/F_m_ was significantly greater. This implied that the *gpt2* mutants experienced more photoinhibition of PSII. Nevertheless, no clear growth phenotype difference was observed except for a minor discolouration at the edge line of the mature leaves, a sign of anthocyanin accumulation in the mutant (Supplementary Figure 1).

The exposure of leaves to excess light can increase the formation of ROS. Amongst the primary targets for damage by such ROS are membrane lipids. Oxidation of lipids can lead, amongst other products, to the formation of the relatively stable molecule MDA. Malondialdehyde accumulation is, therefore, commonly used as an indicator for oxidative stress. The leaves of the Ws-4 and *gpt2* plants at control light or HL were flash-frozen under growth conditions and assayed for anthocyanin content. In the Ws-4 WT, exposure to HL did not result in an increase in leaf MDA content (Figure 2B). In contrast, in the *gpt2* mutant, a significant increase in MDA was observed.

### Plants Lacking GPT2 Induce Greater Amounts of Antioxidant Enzymes but Less Ascorbate in High Light

**Figure 3:**
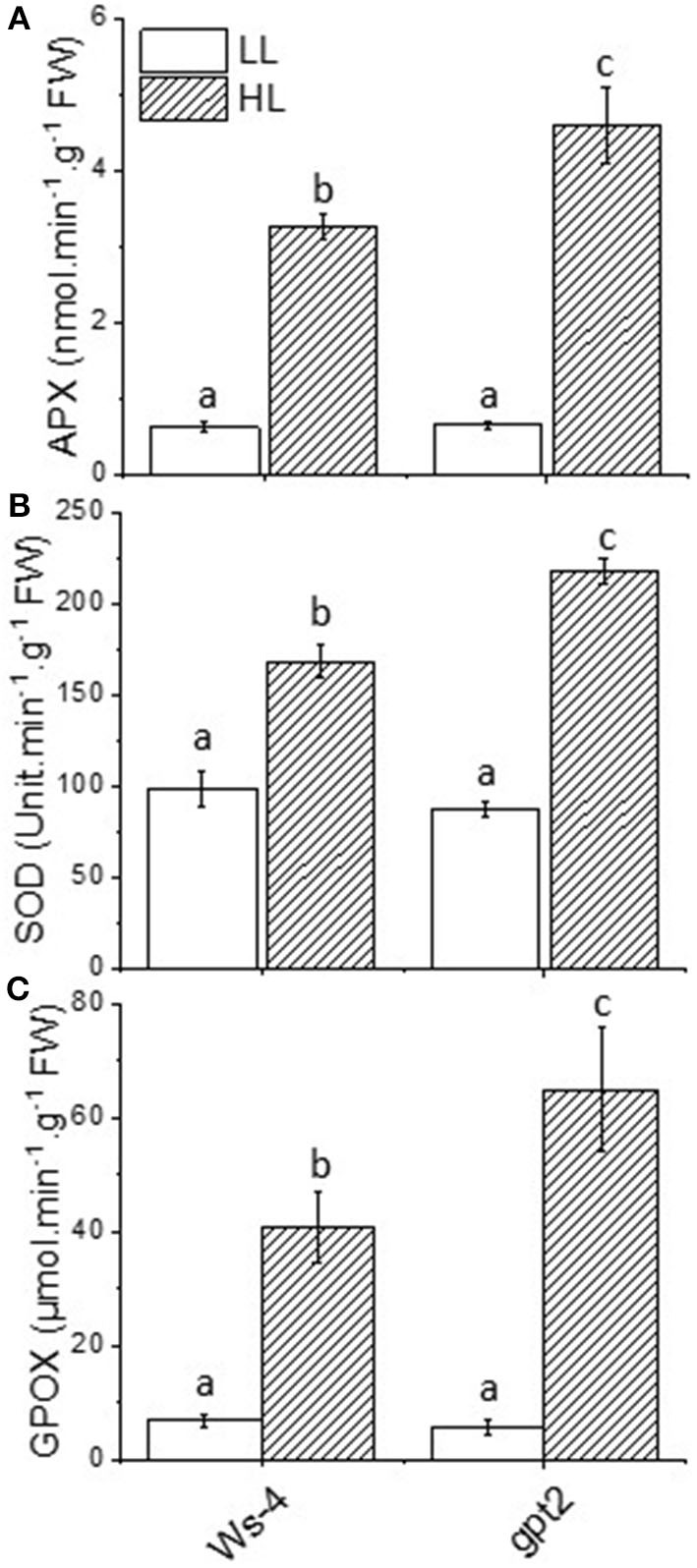
Antioxidant enzyme activities in Ws-4 wild type and *gpt2* plants. Enzymatic activity of ascorbate peroxidase (APX) **(A)**, superoxide dismutase (SOD) **(B)**, and guiacol peroxidase (GPOX) **(C)** in plants that were grown for 7 weeks at 100 μmol m^−2^s^−1^ light and then either maintained at 100 μmol m^−2^s^−1^ for 7 more days (LL) or transferred to conditions with an irradiance of 400 μmol m^−2^s^−1^ (HL). Samples were collected at mid-day on Day 7 of the treatment. The data were analysed using a two-way ANOVA followed by Tukey's test at *p* ≤ 0.05 to be significantly different. Results are *M* ± *SE* of 6–9 independent replicates. Different lowercase letters denote significantly different values.

Electron flow to oxygen, which leads to the formation of superoxide, is liable to occur when reduced electron transport carriers accumulate, in particular Fe-containing centres such as the FeS clusters on the acceptor side of PSI. It has been shown that the careful regulation of electron flow to PSI is essential to avoid oxidative stress (Johnson et al., 2014). Thus, superoxide formed in this way is detoxified by antioxidant pathways such as the Mehler-ascorbate peroxidase (MAP) pathway. The key enzymes involved in detoxifying superoxide include SOD, APX, and GPX. Thus, to assess the impact of HL on these systems, the total leaf activity of SOD, APX, and guiacol peroxidase (GPOX) were measured. Under LL conditions, no difference was observed between Ws-4 WT and *gpt2* in any of these activities (Figures 3A–C). However, when WT plants were exposed to HL for 7 days, the activities of SOD, APX, and GPX all increased significantly. An increase was also seen in *gpt2* plants, but the activity attained was greater in all cases than that seen in Ws-4.

**Figure 4:**
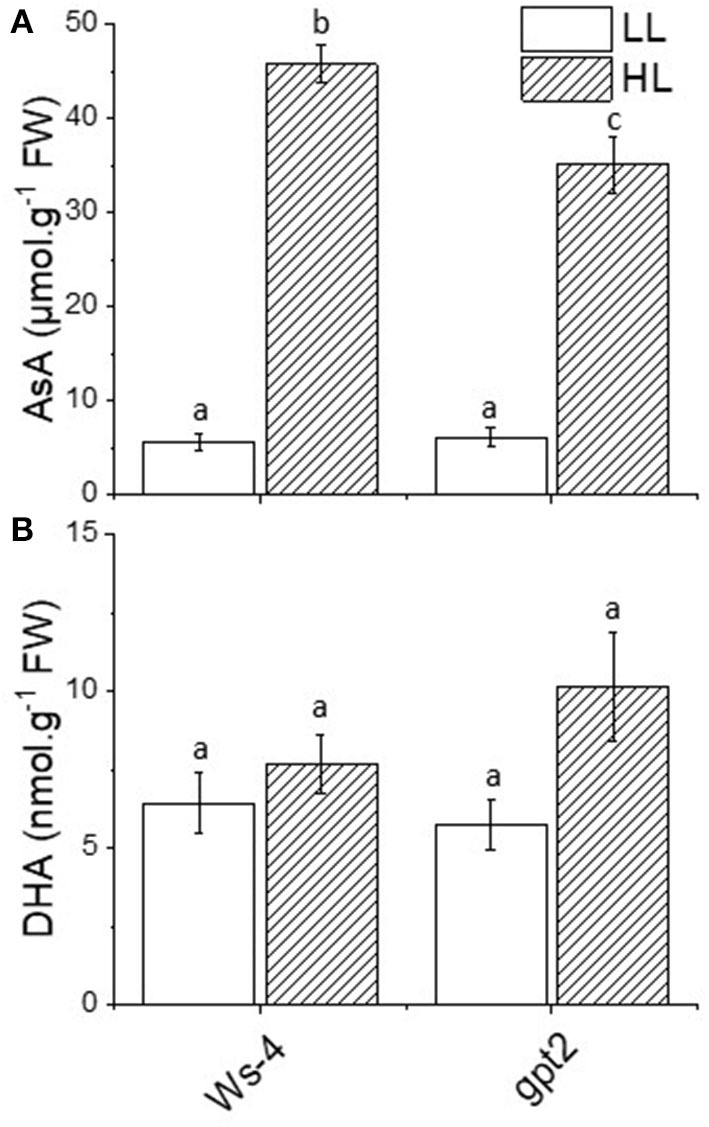
Antioxidant content of the Ws-4 wild type and *gpt2* plants. The leaf content of ascorbic acid (AsA0) **(A)** and dehydroascorbate (DHA) **(B)** in plants that were grown for 7 weeks at 100 μmol m^−2^s^−1^ light and then either maintained at 100 μmol m^−2^s^−1^ for 7 more days (LL) or transferred to conditions with an irradiance of 400 μmol m^−2^s^−1^ (HL). Leaves were flash-frozen under growth conditions. Extractions and measurements were done on the same day to minimise the rapid oxidation of AsA to DHA in the sample. The data were analysed using a two-way ANOVA followed by Tukey's test at *p* ≤ 0.05 to be significantly different. Different lowercase letters denote significantly different.

The activity of APX relied on the presence of ascorbate. In response to HL, the leaf content of ascorbate increased significantly and substantially in both Ws-4 and *gpt2*. However, the concentration was higher in acclimated Ws-4 WT than in the mutants (Figure 4A). When ascorbate was used to detoxify reactive O, DHA was produced as a product, but concentrations of oxidised DHA did not vary significantly between plants or in response to HL (Figure 4B).

**Figure 5:**
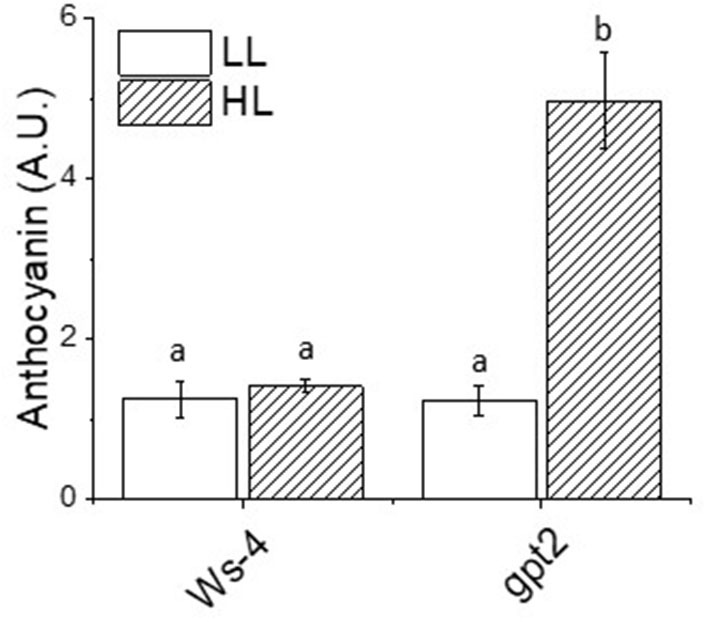
Relative anthocyanin content of the Ws-4 wild type and *gpt2* plants. Plants were grown for 7 weeks at an irradiance of under 100 μmol m^−2^ s^−1^ before either remaining at that irradiance (LL) or being transferred to 400 μmol m^−2^ s^−1^ (HL) conditions for 7 more days. Leaf samples were collected at the end of the photoperiod and flash-frozen prior to extraction. The data were statistically analysed using a two-way ANOVA followed by Tukey's test at *p* ≤ 0.05 to be significantly different. Values represent the *M* ± *SE* of *n* = 5. Different letters above bars indicate significantly different values.

In addition to containing high concentrations of antioxidants specifically associated with the MAP pathway, the plants also accumulated an array of other compounds with antioxidant activity. This includes the notable and most obvious anthocyanins, which give leaves their obvious red-purple colour. However, the leaves of Ws-4 under control conditions contained low amounts of anthocyanin, and the transfer to HL did not increase this significantly (Figure 5). Under control conditions, *gpt2* did not differ significantly from Ws-4, but following the exposure to HL, anthocyanin content rose significantly and substantially.

### Transcriptomics and Proteomics Reveal the Elevated Expression of a Wide Range of Stress-Linked Genes

Previous studies of the responses of the *gpt2* and Ws-4 plants to moderate increases in light, from 100 to 400 μmol.m^−2^s^−1^ as used here, revealed little evidence for the increases in transcript levels of stress-related genes in either genotype following short-term (4 h) exposure to HL (Athanasiou et al., 2010; Dyson et al., 2015). This implies that the treatment used did not result in an acute stress response. To determine whether longer-term HL exposure induced chronic responses in gene expression, we performed a microarray analysis following 7 days of exposure to HL. In this analysis, the leaf samples were flash-frozen under growth conditions and RNA was extracted. Genes were then seen to be differentially expressed in the WTHL (407), *gpt2*HL (329), and *gpt2*LL (21) relative to the control WTLL (Supplementary Figure 2; for a full list of significantly altered genes, see Supplementary Table 1). In WTHL, there were 177 transcripts upregulated and 230 transcripts downregulated. Meanwhile, in *gpt2HL* plants, 141 genes were increased significantly in response to HL and 188 transcripts repressed. Comparing genotypes at LL, *gpt2*LL had 15 upregulated genes with only 6 being repressed (Supplementary Figure 2). Out of a total of 318 transcripts upregulated in both genotypes at HL, 92 of them were similar, 83 were only seen in WT, and 47 genes were seen in *gpt2*. Besides that, there was only one gene that appeared in all plants and one gene upregulated in both the *gpt2*HL and *gpt2*LL (Supplementary Figure 2). Out of a total of 418 downregulated transcripts, 126 genes were common to WTHL and *gpt2*HL, 101 transcripts were unique to WTHL, and 60 transcripts were unique to *gpt2HL*. Meanwhile, three genes were repressed only in *gpt2*LL, another one gene was downregulated in both WTHL and gpt2LL, and two genes were in all WTHL, *gpt2*HL, and *gpt2*LL plants.

**Figure 6:**
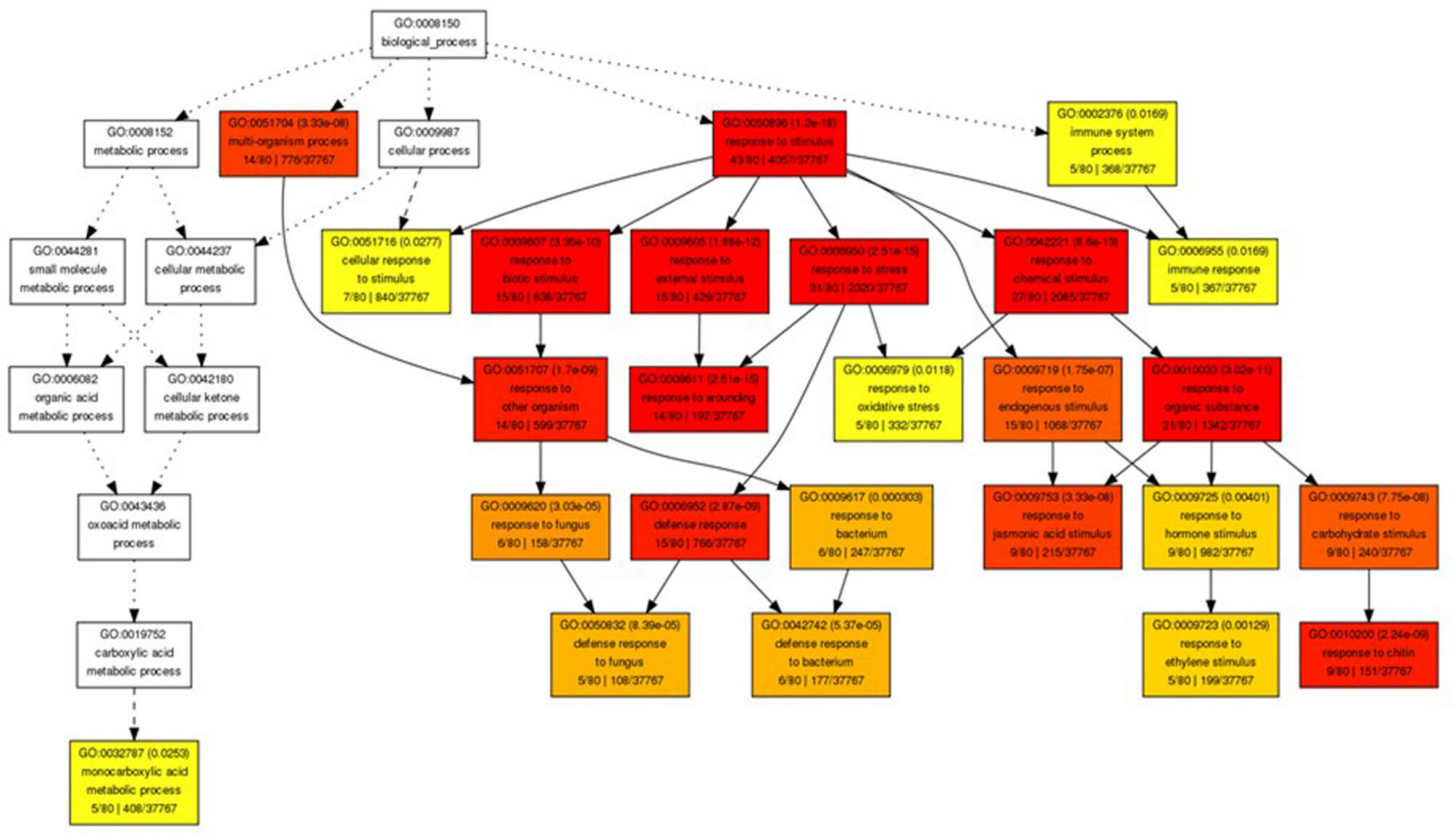
Gene ontology (GO) analysis of genes with altered expression in Ws-4 wild type and *gpt2* plants. Graphical representation of a GO hierarchical image containing all statistically significant terms between *gpt2*HL/WTHL at Day 7. These nodes in the image are classified with specific corresponding colours. The smaller the adjusted *p*-value of the term, the more significant statistically, and the darker and redder the colour of the node. Inside the box of the significant terms, the information includes the GO term, adjusted *p*-value, GO description, item number mapping the GO in the query list and background, and the total number of query list and backgrounds. Image produced using online GO Analysis Toolkit and Database for Agricultural Community (http://bioinfo.cau.edu.cn/agriGO/analysis.php).

The gene ontology analysis of the upregulated genes in the *gpt2*HL/WTHL at Day 7 showed that most transcripts were categorised in “response to stimulus” (Figure 6). A further breakdown of the node to see the specific annotation of the transcripts developed another six GO terms including “response to biotic, external stimulus,” “stress,” and “chemical stimulus,” which were highly significant according to the *p*-value and darker colour of the nodes. There were only five genes specifically classified under “oxidative stress:” AT1G19020 (small defence-associated protein 1), AT3G16670 (allergen and extension family protein pollen Ole e 1), AT1G27730 (salt tolerance zinc finger ZAT10), AT1G02930 (glutathione S-transferase 1), and AT5G20230 (blue-copper-binding protein). Nevertheless, this should not be a definite number of genes involved in oxidative stress in this analysis, since most stresses, including wounding and biotic responses (in separate nodes), would also cause imbalances in redox states. In fact, the overall view of the hierarchical GO shows that most of the nodes or the GO terms have relationships to stress according to the available literature. In addition, to look for the GO terms of the genes that were upregulated in WTHL against *gpt2*, a reverse comparison was done between WTHL and *gpt2*HL. However, the results showed that no significantly enriched GO terms were identified. This observation showed that the induction of a high number of stress-related genes in *gpt2* plants suggested that they experienced more stress at HL than in the WT.

## Discussion

The overall aim of this work was to examine the importance of photosynthetic acclimation in protecting plants from abiotic stress, focusing on increases in light intensity. An important protein controlling the acclimation process is GPT2, as plants lacking this protein have impaired photosynthetic acclimation to light (Athanasiou et al., 2010). Since this discovery, the investigation of the importance of this protein in acclimation to HL has continued, with a series of studies looking at the different aspects of this finding using metabolomic and proteomic approaches (Dyson et al., 2014, 2015; Miller et al., 2017). Importantly, these studies showed that there is a significant cost when there is a failure to acclimate in terms of fitness and seed yield. In part, this was explained by a decrease in photosynthesis. However, the results presented by this current study indicated that there is also a likely additional cost in terms of the extent to which plants experience oxidative stress.

In this study and the previous work leading to it, irradiance conditions that did not represent substantial stress to plants were selected. Most previous studies of HL stress employed considerably higher, often non-physiological, irradiances, with the intention of inducing severe stress events in plants (Aarti et al., 2007; Zuluaga et al., 2008; Galvez-Valdivieso et al., 2009; Bayat et al., 2018; Maai et al., 2020). In this study, we not only maintained the maximum photosynthetic capacity during HL, but we also had strong evidence that photoacclimation could alleviate the stress effect in plants (Athanasiou et al., 2010; Dyson et al., 2014). This can be seen both in the measurements of stress markers and from the analysis of gene expression. The transcriptomic analysis also showed that the expression of stress-related genes increased in both the WT and mutant plants in response to HL. However, the responses linked to oxidative stress were more pronounced in the *gpt2* plants than WT.

Light is not only an important factor in photosynthesis, but it also mediates signals to control plant development and growth and induces stress tolerance (Spetea et al., 2014). In the present work, we saw that gene expression profiles differed in the *gpt2* and Ws-4 plants following their exposure to HL. This is consistent with the previous observation that these plants showed different metabolomic and proteomic responses to sustained HL (Dyson et al., 2015; Miller et al., 2017. This could be seen in the GO analysis (Figure 6) where the *gpt2*HL had more transcripts in “response to stimulus,” while hormonal genes were also induced. This was supported in an earlier study conducted by Miller et al. (2017) that showed that *gpt2* plants invested more in stress-related proteins, suggesting that their reduced ability to increase P_max_ could result in increased stress. Meanwhile, the increases in protein expression related to enzymatic activity in the Calvin–Benson cycle were observed in the WT plants, resulting in a higher P_max_, and concomitantly showing that acclimation could avoid significant stress incidence in plants (Miller et al., 2017).

There was no evidence for the increases in the transcript levels of genes encoding proteins involved in photosynthesis at Day 7 in the microarray, which is consistent with previous observations at Days 1 and 3 of the treatment (Athanasiou et al., 2010). Nonetheless, we did find that several genes upregulated only in the WTHL and WTLL were associated with the chloroplast (APO1, Peptidase S41 family protein, and FTSH8). These genes were neither involved directly in the photosynthetic apparatus nor scavenging ROS, but may increase plant fitness by organising PSII repair, preventing cell death, or promoting the accumulation of PSI and NADH dehydrogenase complexes (Watkins et al., 2011; Lu, 2016). In other words, the steps taken in the WT plants were done mostly to improve chloroplast integrity and fitness rather than invest more into metabolically expensive stress-responsive proteins, thus improving tolerance in acclimated plants.

The induction of glucose-6-phosphate/phosphate translocator 2 in the chloroplast membrane to maintain the homeostasis between plastids and cytosols may also increase fitness in the chloroplast and indirectly protect the cell components and DNA from damage during an HL treatment. There are four important sugar-phosphate/phosphate translocator families located in the chloroplast envelope (Knappe et al., 2003), but among these, the requirement for the GPT2 in plants acclimated to HL was very clear (Athanasiou et al., 2010). The other three translocators, namely, the triose phosphate translocator (TPT), phosphoenolpyruvate translocator (PPT), and xylulose-5-phosphate translocator (XPT), did not appear to be significantly induced at a transcriptomic level on either Day 1, based on the study of Athanasiou et al. (2010), or Day 7 in the WT, based on this study. Interestingly, the mutant also did not seem to compensate for the GPT2 deficiency, showing no increases in TPT, PPT, or XPT. Thus, the lack in GPT2 blocked the translocation of glucose-6-phosphate, a sole precursor for starch synthesis in most plants, which subsequently impaired the metabolism in the chloroplasts (Harrison et al., 2000; Kofler et al., 2000). Although there was a chance that the photoassimilate could be directly diverted into starch, this chance was small (Walters et al., 2004).

The physiological approaches to measuring plants stress were easily assessed by looking at the levels of F_v_/F_m_ between the WT and *gpt2*. Significant reductions in F_v_/F_m_ in the mutant could be an early indicator of an inability to acclimate to excessive light-caused photoinhibition, targeting mainly the PSII core complex. The results of our previous P_max_ measurements confirmed this, with less capacity seen in the *gpt2* compared with WT. Although having a similar amount of total chlorophyll, the WT had a lower Chl *a*:*b* at HL, which is consistent with a higher number of reaction centre chlorophylls. This was relative to the antenna; thus, less energy was captured per reaction centre that avoided too much pressure on the photosynthetic electron transport chain.

High light stress is mainly due to the overproduction of ROS, leading to damage to the photosynthetic apparatus (Aro et al., 1993). In the present study, antioxidant enzyme activities were induced at HL, but the induction was greater in the *gpt2* plants. This is a clear indication that the failure to acclimate to HL could cause more oxidative stress, thus requiring higher enzyme activities to counteract ROS and prevent damage. Although several studies have reported that acclimation improved light stress tolerance, this may be partially attributed to the high levels of radical scavengers, including antioxidants and antioxidant enzyme activity (Close and Mcarthur, 2002; Ali et al., 2005; Agati and Tattini, 2010), but this depends on the state of the cells or tissues and responses may vary (Costantini and Verhulst, 2009). Based on such observations, we suggested that the lower activities of antioxidant enzymes seen at HL in the WT plants were primarily due to an ability to acclimate dynamically to the changes in light intensity. Therefore, there would be less ROS produced and lower antioxidant enzymes activities induced. The observation that the AsA levels were lower in the *gpt2* plants at HL than in the Ws-4 was perhaps surprising in this context. We suggested that this may reflect the greater turnover of AsA because the observation that DHA tends to be higher in the gpt2 at HL was consistent with this, but this effect was not statistically significant. The increased accumulation of anthocyanins in the *gpt2* plants at HL, however, was consistent with plants experiencing greater stress. Furthermore, no genes known to be involved in anthocyanin synthesis showed increased transcript levels, indicating that this increase in accumulation must have resulted from either the post-transcriptional regulation of protein expression or the increased activity of enzymes.

Extended exposure to HL induced higher enzyme activities, which could indicate that plants became more sensitive due to increases of ROS in the cell. Oxidative stress would induce the regulation of ROS-dependent genes at the transcription level, especially in antioxidant enzymes, until both could reach the equilibrium (Mylona and Polidoros, 2010). In the present work, the effect of HL on membrane lipids on the production of MDA in the leaves can be seen from our results. Despite having higher antioxidant activities and metabolic rates at HL, no sign of increased membrane damage was observed in the WTHL compared with control LL. This could be viewed as a result of a very efficient action by antioxidant enzymes. According to a study by Dong et al. (2016), acclimation can protect plants by altering their membrane compositions and increasing membrane integrity. Therefore, this result supports our hypothesis that acclimation prevents oxidative stress and improves plant tolerance to HL. Meanwhile, the increased lipid peroxidation in *gpt2* plants during acclimation to HL is a clear indication that they had reduced abilities to tolerate HL, thus making their membranes more vulnerable to ROS.

Although the glucose-6-phosphate/phosphate translocator 2 protein has been well-reported, the function of this protein in stress is not yet known. Current evidence did not explicitly explain how GPT2 would have a direct effect on oxidative stress protection in plants. However, it was reported that G6P, the homeostasis in which between cytosol and chloroplast is probably controlled by GPT2, also takes part in regulatory mechanisms in response to stress (Dobrenel et al., 2013). Increased G6P concentrations not only promoted starch synthesis, but could also trigger G6P dehydrogenase to funnel the NADPH-producing metabolism in the oxidative pentose phosphate pathway (OPPP) in response to stress (Couée et al., 2006; Bolouri-Moghaddam et al., 2010). Recently, a study by Weise et al. (2019) also suggested that when photosynthesis rises, GPT2 may operate as a safety valve by diverting sugar phosphates away from the non-phosphorylating GAPDH in the cytosol, which could be important for regulating and stabilising photosynthetic electron transport and carbon metabolism in chloroplasts, something that is lacking in the mutants. Meanwhile, the mutant also did not seem to compensate for the GPT2 deficiency, showing no increases in the expression of other sugar translocators in this study. The failure to translocate G6P, a precursor for starch synthesis, subsequently impairs metabolism in the chloroplasts (Harrison et al., 2000; Kofler et al., 2000).

## Conclusion

The analysis of the transcriptomic data in both the WT and *gpt2* mutant plants supported our hypothesis that plants that are unable to acclimate to HL experience increased oxidative stress. While neither the genes involved in the photosynthetic electron transport chain nor in ROS scavenging were upregulated, a few genes associated with the chloroplast did show significantly increased transcript levels. The above transcriptomic microarray analysis findings are consistent with our physiological and biochemical evidence, showing that the GPT2 protein is essential for photosynthetic acclimation in *A. thaliana* and could prevent the severe effects of HL stress.

## Data Availability Statement

The datasets presented in this study can be found in online repositories. The names of the repository/repositories and accession number(s) can be found at: https://www.ebi.ac.uk/arrayexpress/, E-MTAB-10282.

## Author Contributions

MFK and GJ designed the experiments and wrote the paper. MFK performed the experiments and analysed the results.

## Funding

MFK was supported by a studentship from the Ministry of Higher Education of Malaysia and the International Islamic University Malaysia.

## Conflict of Interest

The authors declare that the research was conducted in the absence of any commercial or financial relationships that could be construed as a potential conflict of interest.

## Publisher's Note

All claims expressed in this article are solely those of the authors and do not necessarily represent those of their affiliated organizations, or those of the publisher, the editors and the reviewers. Any product that may be evaluated in this article, or claim that may be made by its manufacturer, is not guaranteed or endorsed by the publisher.

## Abbreviations

APX: Ascorbate peroxidase
AsA: ascorbic acid
DHA: dehydroascorbic acid
GPT2: glucose-6-phosphate/phosphate translocator 2
GPX: glutathione peroxidase
HL: high light
LL: low light
${\text{O}}_{2}^{-}$: superoxide
^1^ ${\text{O}}_{2}^{*}$: singlet excited oxygen
PSI: photosystem I
PSII: photosystem II
ROS: reactive oxygen species
SOD: superoxide dismutase.
