# Supplementary figures and images for: Acclimation of Photosynthesis to Changes in the Environment Results in Decreases of Oxidative Stress in Arabidopsis thaliana
Source: Front Plant Sci. 2021 Sep 23;12:683986. doi: 10.3389/fpls.2021.683986 (PMC8495028; doi:10.3389/fpls.2021.683986)

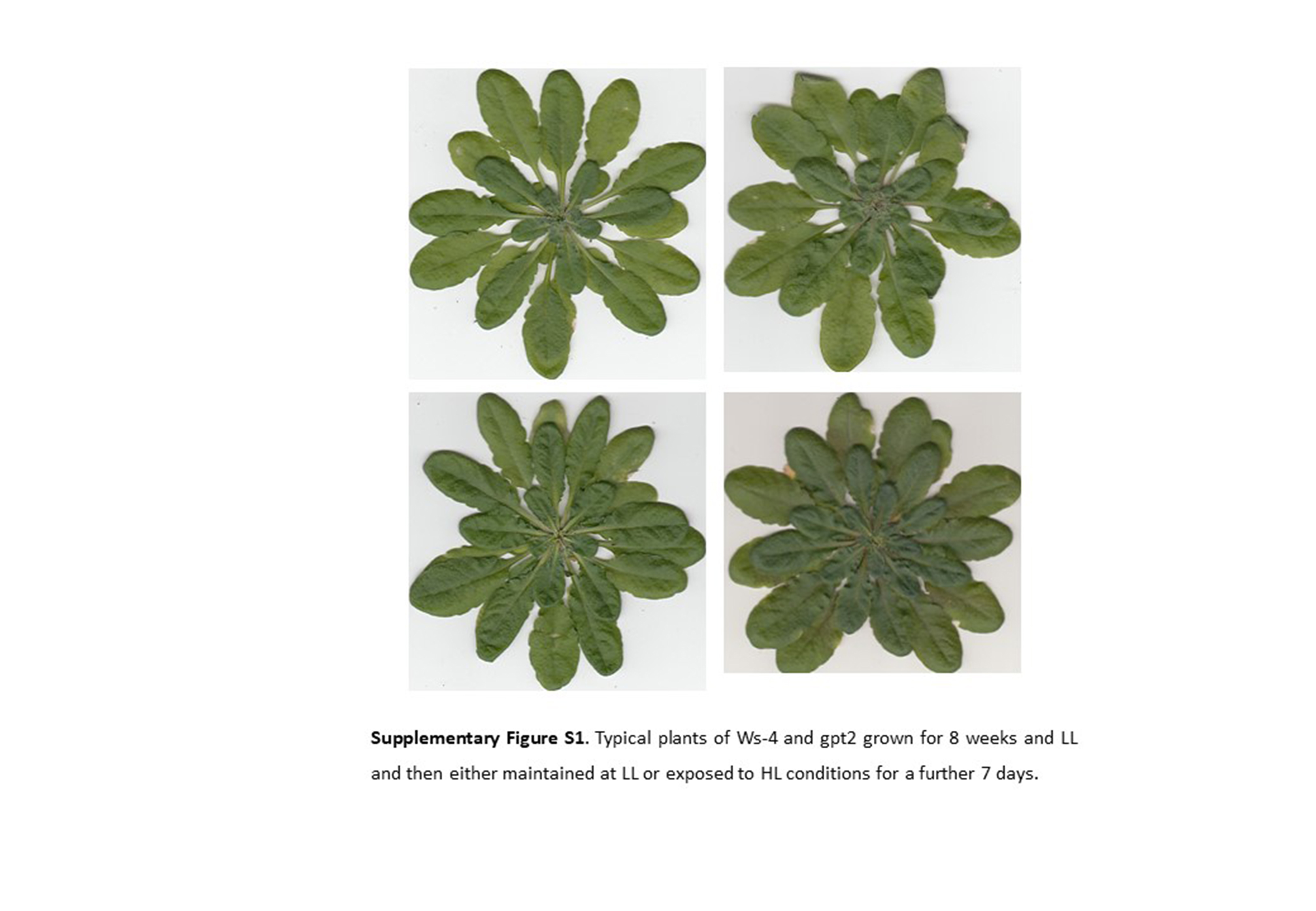

Supplement: Supplementary file 1 [file Image_1.JPEG]

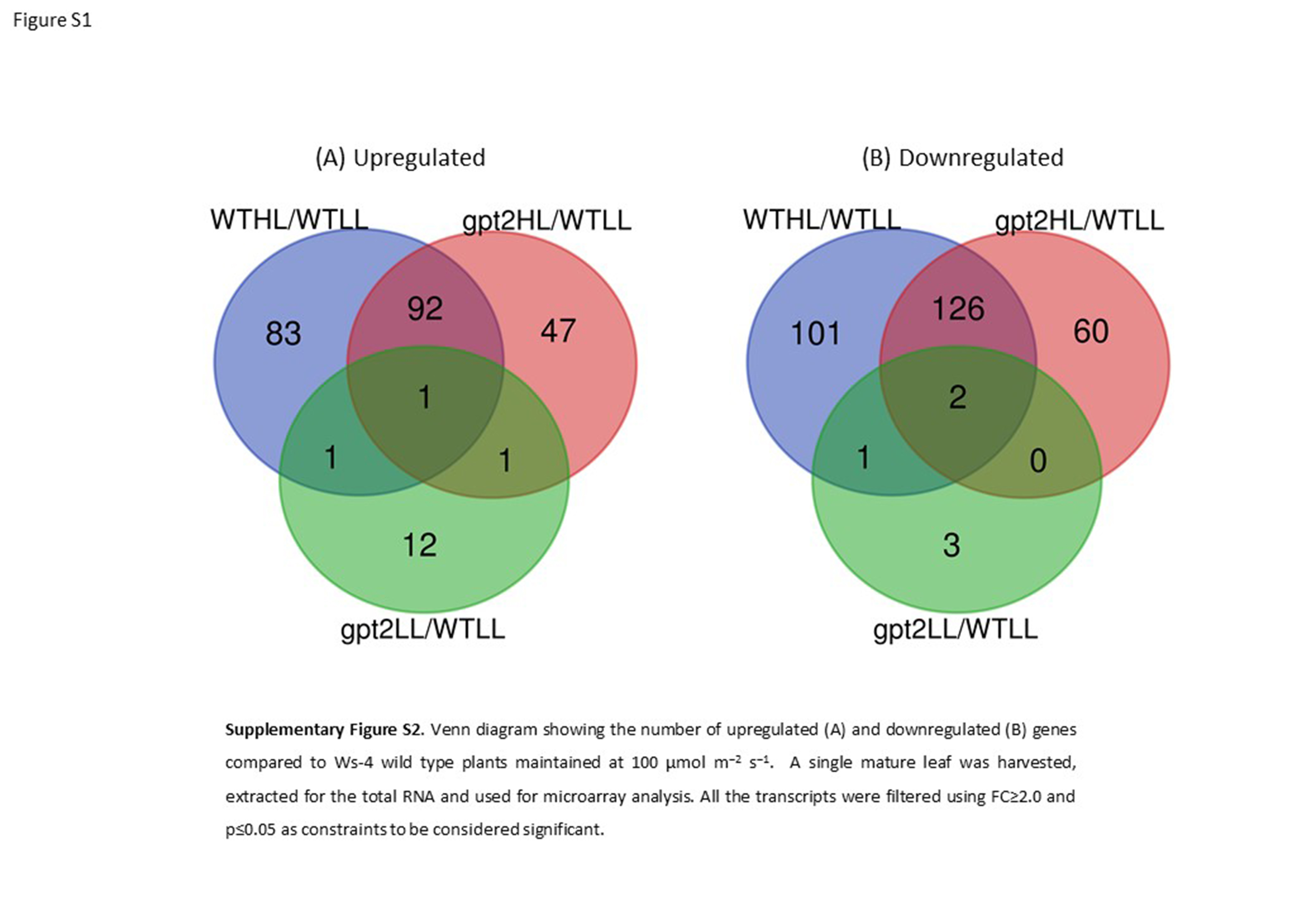

Supplement: Supplementary file 2 [file Image_2.JPEG]
