# Supplementary material for: Acclimation of Photosynthesis to Changes in the Environment Results in Decreases of Oxidative Stress in Arabidopsis thaliana
Source: Front Plant Sci. 2021 Sep 23;12:683986. doi: 10.3389/fpls.2021.683986 (PMC8495028; doi:10.3389/fpls.2021.683986)
Supplement: Supplementary file 3 [file Table_1.DOCX]

**Acclimation of photosynthesis to changes in the environment results decreases oxidative stress in *Arabidopsis thaliana***

Mohd Fauzihan Karim^*^ and Giles N Johnson^#^

University of Manchester, School of Earth and Environmental Sciences, Michael Smith Building, Oxford Rd, Manchester, M13 9PT

^*^Current address: Department of Plant Science, Kulliyyah of Science, International Islamic University Malaysia, 25200 Kuantan, Malaysia; email: mfauzihan@iium.edu.my

**TABLE OF CONTENT**

|  | **ELEMENTS** | **PAGE** |
| --- | --- | --- |
| 1 | WTHL / WTLL - Upregulated genes list | 2 |
| 2 | WTHL / WTLL - Downregulated genes list | 11 |
| 3 | *gpt2*HL / WTLL - Upregulated genes list | 24 |
| 4 | *gpt2*HL / WTLL - Downregulated genes list | 31 |
| 5 | *gpt2*LL / WTLL - Upregulated genes list | 42 |
| 6 | *gpt2*LL / WTLL - Downregulated genes list | 43 |

**WTHL/WTLL**

***UPREGULATION***

| **Transcript ID** | **Target Description** | **Fold Change** |
| --- | --- | --- |
| At4g15210 | beta-amylase ; supported by cDNA: gi_166601_gb_M73467.1_ATHAMYB | 29.2086 |
| At3g15650 | putative lysophospholipase similar to lysophospholipase GB:AAD52700 [Schistosoma jap onicum] | 20.7511 |
| At5g59320 | nonspecific lipid-transfer protein precursor - like nonspecific lipid-transfer protein precursor, Brassica napus, EMBL:AF101038;supported by full-length cDNA: Ceres:7828. | 19.3048 |
| At5g59310 | nonspecific lipid-transfer protein precursor - like nonspecific lipid-transfer protein precursor, Brassica napus, EMBL:AF101038;supported by full-length cDNA: Ceres:43057. | 17.7144 |
| At5g24780 | vegetative storage protein Vsp1 ;supported by full-length cDNA: Ceres:32606. | 17.0076 |
| At5g17220 | glutathione S-transferase-like protein ; supported by cDNA: gi_11096011_gb_AF288189.1_AF288189 | 13.7708 |
| At1g56650 | anthocyanin2, putative similar to anthocyanin2 (An2) GI:7673088 from [Petunia integrifolia]; supported by cDNA: gi_3941507_gb_AF062908.1_AF062908 | 12.8954 |
| At1g61800 | glucose-6-phosphate/phosphate-translocator precursor, putative similar to glucose-6-phosphate/phosphate-translocator precursor GI:2997591 from [Pisum sativum]; supported by cDNA: gi_14596172_gb_AY042874.1_ | 12.7357 |
| At4g22870 | anthocyanidin synthase - like protein putative leucoanthocyanidin dioxygenase, Arabidopsis thaliana, PID:g1575699 | 10.4192 |
| At5g13930 | chalcone synthase (naringenin-chalcone synthase) (testa 4 protein) (sp\|P13114) ;supported by full-length cDNA: Ceres:38370. | 10.4146 |
| At3g16360 | putative two-component phosphorelay mediator similar to two-component phosphorelay mediators (ATHP1-3) GB:BAA37110, GB:BAA37111, GB:BAA37112 (Arabidopsis thaliana); supported by cDNA: gi_7630186_dbj_AB041766.1_AB041766 | 10.1714 |
| At5g42800 | dihydroflavonol 4-reductase | 9.42015 |
| At5g58770 | dehydrodolichyl diphosphate - like protein dehydrodolichyl diphosphate, Arabidopsis thaliana, EMBL:ATH277136 | 9.37128 |
| At4g14090 | glucosyltransferase like protein | 8.44003 |
| At4g23990 | cellulose synthase catalytic subunit - like protein cellulose synthase catalytic subunit (Ath-A), Arabidopsis thaliana, gb:AF027173 | 8.13066 |
| At1g03495 | hypothetical protein similar to Anthocyanin 5-aromatic acyltransferase GB:BAA74428 | 8.10591 |
| At3g08860 | putative aminotransferase similar to beta-alanine-pyruvate aminotransferase GB:BAA19549 [Rattus norvegicus], alanine-glyoxylate aminotransferase GB:Q64565 [Rattus norvegicus]; Pfam HMM hit: Aminotransferases class-III pyridoxal-phosphate | 8.01627 |
| At2g40100 | putative chlorophyll a/b binding protein ; supported by full-length cDNA: Ceres: 6454. | 7.51002 |
| At2g21590 | putative ADP-glucose pyrophosphorylase large subunit | 6.77548 |
| At1g73040 | jacalin, putative similar to jacalin GI:289164 from [Artocarpus heterophyllus] | 6.64489 |
| At5g19470 | putative protein thiamin pyrophosphokinase, Schizosaccharomyces pombe, PIR:S52350 | 6.44113 |
| At5g11930 | glutaredoxin - like protein glutaredoxin, R.communis, EMBL:RCGLREDXN | 6.09758 |
| At1g32900 | starch synthase, putative similar to starch synthase GI:21613 from [Solanum tuberosum];supported by full-length cDNA: Ceres:7714. | 6.02604 |
| At1g34040 | allinase, putative similar to allinase GI:1044969 from [Allium cepa] | 5.58418 |
| At4g39210 | glucose-1-phosphate adenylyltransferase (APL3) ; supported by cDNA: gi_16648984_gb_AY059862.1_ | 5.56112 |
| At2g17710 | unknown protein ;supported by full-length cDNA: Ceres:1011. | 4.99275 |
| At4g16590 | cellulose synthase like protein | 4.86142 |
| At2g37760 | putative alcohol dehydrogenase ; supported by cDNA: gi_16604706_gb_AY059798.1_ | 4.8356 |
| At1g62710 | beta-VPE nearly identical to beta-VPE GB:BAA09615 GI:1805364 [Arabidopsis thaliana]; supported by cDNA: gi_14194096_gb_AF367254.1_AF367254 | 4.82557 |
| At2g25625 | Expressed protein ; supported by full-length cDNA: Ceres: 465. | 4.5899 |
| At1g02850 | beta-glucosidase, putative similar to beta-glucosidase GI:5030906 from [Polygonum tinctorium]; supported by cDNA: gi_15146265_gb_AY049274.1_ | 4.58622 |
| At1g21400 | branched-chain alpha keto-acid dehydrogenase, putative similar to branched-chain alpha keto-acid dehydrogenase GB:AAC69851 GI:3822223 from [Arabidopsis thaliana] | 4.55796 |
| At1g77920 | transcription factor, putative similar to transcription factor GI:304113 from [Arabidopsis thaliana]; supported by cDNA: gi_16209662_gb_AY057596.1_ | 4.53972 |
| At1g64780 | ammonium transporter, puitative similar to ammonium transporter GI:5880357 from [Arabidopsis thaliana]; supported by cDNA: gi_4324713_gb_AF110771.1_AF110771 | 4.31098 |
| At3g49580 | putative protein ;supported by full-length cDNA: Ceres:26235. | 4.16731 |
| At1g57590 | pectinacetylesterase precursor, putative similar to pectinacetylesterase precursor GI:1431629 from [Vigna radiata] | 4.14737 |
| At1g67360 | stress related protein, putative similar to stress related protein GI:5802955 from [Vitis riparia];supported by full-length cDNA: Ceres:14043. | 4.03412 |
| At2g29090 | putative cytochrome P450 | 3.96558 |
| At3g22840 | early light-induced protein identical to early light-induced protein GB:AAB88391 from [Arabidopsis thaliana];supported by full-length cDNA: Ceres:14490. | 3.90785 |
| At5g37980 | quinone oxidoreductase -like protein probable quinone oxidoreductase P1, Arabidopsis thaliana;supported by full-length cDNA: Ceres:116237. | 3.8468 |
| At1g52770 | putative non-phototropic hypocotyl similar to non-phototropic hypocotyl 3 GB:AAF05914 from [Arabidopsis thaliana] | 3.82043 |
| At1g19640 | floral nectary-specific protein, putative similar to GB:AAF22289 from [Brassica rapa subsp. pekinensis] (Plant Mol. Biol. 42 (4), 647-655 (2000)); supported by cDNA: gi_13676828_gb_AY008434.1_ | 3.71849 |
| At4g34590 | bZIP transcription factor ATB2 | 3.50388 |
| At5g64550 | putative protein strong similarity to unknown protein (emb\|CAB89363.1) | 3.47428 |
| At3g28270 | unknown protein similar to At14a protein GB:AAD26355 GI:4589123 [Arabidopsis thaliana]; supported by cDNA: gi_16226582_gb_AF428437.1_AF428437 | 3.46626 |
| At5g46830 | bHLH transcription factor | 3.46431 |
| At5g47330 | palmitoyl-protein thioesterase precursor-like | 3.45425 |
| At1g80130 | unknown protein ;supported by full-length cDNA: Ceres:35675. | 3.43702 |
| At4g33040 | putative protein AT.I.24, Arabidopsis thaliana, gb:U63815;supported by full-length cDNA: Ceres:4868. | 3.33077 |
| At5g54060 | flavonol 3-O-glucosyltransferase-like | 3.30081 |
| At1g22160 | unknown protein ;supported by full-length cDNA: Ceres:23788. | 3.26386 |
| At3g51240 | flavanone 3-hydroxylase (FH3) ;supported by full-length cDNA: Ceres:36653. | 3.21548 |
| At2g43580 | putative endochitinase | 3.1682 |
| At2g18230 | putative inorganic pyrophosphatase ;supported by full-length cDNA: Ceres:8068. | 3.16629 |
| At1g55760 | hypothetical protein predicted by genemark.hmm | 3.15797 |
| At3g12580 | heat shock protein 70 identical to heat shock protein 70 GB:CAA05547 GI:3962377 [Arabidopsis thaliana]; supported by cDNA: gi_15809831_gb_AY054183.1_ | 3.10917 |
| At3g57260 | beta-1,3-glucanase 2 (BG2) (PR-2) ;supported by full-length cDNA: Ceres:21719. | 3.10335 |
| At1g78440 | gibberellin 2- oxidase identical to gibberellin 2- oxidase GI:4678366 from [Arabidopsis thaliana];supported by full-length cDNA: Ceres:156482. | 3.08018 |
| At4g37150 | hydroxynitrile lyase like protein | 3.05858 |
| At2g27420 | cysteine proteinase contains similarity to cysteine protease SPCP1 GI:13491750 from [Ipomoea batatas] | 3.04617 |
| At4g17770 | trehalose-6-phosphate synthase like protein ;supported by full-length cDNA: Ceres:95947. | 3.0329 |
| At1g11700 | unknown protein ESTs gb\|R65381 and gb\|T44635 come from this gene | 3.01524 |
| At5g53420 | putative protein similar to unknown protein (pir\|\|T02891);supported by full-length cDNA: Ceres:112574. | 2.99128 |
| At5g48850 | putative protein similar to unknown protein (gb\|AAC72543.1) | 2.95915 |
| At5g22300 | Nitrilase 4 (sp\|P46011) ; supported by full-length cDNA: Ceres: 6220. | 2.94196 |
| At4g26950 | putative protein other Arabidopsis hypothetical proteins | 2.93962 |
| At5g05270 | putative protein contains similarity to chalcone-flavonone isomerase (chalcone isomerase)supported by full-length cDNA: Ceres:40439. | 2.905 |
| At4g01080 | hypothetical protein | 2.90253 |
| At4g32940 | gamma-VPE (vacuolar processing enzyme) ; supported by cDNA: gi_13877794_gb_AF370160.1_AF370160 | 2.89005 |
| At4g17670 | hypothetical protein ;supported by full-length cDNA: Ceres:6709. | 2.87639 |
| At5g24120 | sigma-like factor (emb CAA77213.1) | 2.83797 |
| At4g12280 | copper amine oxidase like protein (fragment2) copper amine oxidase - Cicer arietinum,PID:e1335964 | 2.77806 |
| At3g58070 | zinc finger-like protein several zinc finger proteins - Arabidopsis thaliana | 2.74928 |
| At4g09030 | arabinogalactan-protein homolog arabinogalactan-protein - Arabidopsis thaliana,PID:g3883126; supported by cDNA: gi_10880496_gb_AF195891.1_AF195891 | 2.73498 |
| At1g15410 | hypothetical protein Is a member of the PF\|01177 Aspartate-glutamate racemase family. EST gb\|T43554 comes from this gene | 2.72256 |
| At2g41410 | calmodulin-like protein identical to GB:X68054;supported by full-length cDNA: Ceres:11537. | 2.70353 |
| At1g75390 | bZIP transcription factor ATB2, putative similar to GB:CAA18838 from [Arabidopsis thaliana] | 2.69679 |
| At1g63840 | putative RING zinc finger protein Pfam HMM hit: zinc finger, C3HC4 type (RING finger); supported by cDNA: gi_16648709_gb_AY058131.1_ | 2.61758 |
| At5g25140 | cytochrome P450-like protein CYTOCHROME P450 71B1 - Thlaspi arvense, EMBL:L24438 | 2.60439 |
| At5g01300 | putative protein several hypothetical proteins - different bacteria | 2.60402 |
| At1g28670 | lipase identical to lipase GB:AAA93262 GI:1145627 [Arabidopsis thaliana] (FEBS Lett. 377 (3), 475-480 (1995)); supported by cDNA: gi_1145626_gb_U38916.1_ATU38916 | 2.6015 |
| At3g53230 | CDC48 - like protein transitional endoplasmic reticulum ATPase, Arabidopsis thaliana, PIR:S60112 | 2.59304 |
| At4g33905 | Expressed protein ; supported by cDNA: gi_13358221_gb_AF325032.2_AF325032 | 2.59202 |
| At5g17050 | UDP glucose:flavonoid 3-o-glucosyltransferase -like protein UDP glucose:flavonoid 3-o-glucosyltransferase, Vitis vinifera, EMBL:AF000372 | 2.58795 |
| At1g31820 | amino acid permease, putative contains Pfam profile: PF00324: Amino acid permease | 2.57485 |
| At4g15490 | indole-3-acetate beta-glucosyltransferase like protein ;supported by full-length cDNA: Ceres:35383. | 2.56616 |
| At2g36885 | Expressed protein ; supported by full-length cDNA: Ceres: 29157. | 2.53596 |
| At3g26290 | cytochrome P450, putative contains Pfam profile: PF00067 cytochrome P450 | 2.5057 |
| At2g36750 | putative glucosyl transferase | 2.4874 |
| At3g23920 | beta-amylase, putative similar to beta-amylase GB:CAB58423 [Arabidopsis thaliana]; supported by cDNA: gi_14194172_gb_AF367293.1_AF367293 | 2.47592 |
| At4g15530 | pyruvate,orthophosphate dikinase | 2.4698 |
| At3g52180 | putative protein ;supported by full-length cDNA: Ceres:114734. | 2.46895 |
| At1g79110 | hypothetical protein predicted by genscan+;supported by full-length cDNA: Ceres:36412. | 2.46789 |
| At1g28570 | lipase, putative contains Pfam profile: PF00657 Lipase/Acylhydrolase with GDSL-like motif | 2.46494 |
| At4g08300 | nodulin-like protein nodulin gene MtN21 - Medicago truncatula, PID:e1172471 | 2.43097 |
| At1g07300 | hypothetical protein predicted by genemark.hmm | 2.42963 |
| At4g23900 | hypothetical protein ;supported by full-length cDNA: Ceres:38500. | 2.40587 |
| At1g61930 | hypothetical protein similar to hypothetical protein GI:2894571 from [Arabidopsis thaliana];supported by full-length cDNA: Ceres:30230. | 2.40038 |
| At4g09020 | isoamylase-like protein probable isoamylase (EC 3.2.1.68) su1 - Zea mays,PIR2:T01321 | 2.39902 |
| At2g46680 | homeodomain transcription factor (ATHB-7) identical to SP:P46897; supported by cDNA: gi_15027938_gb_AY045826.1_ | 2.39668 |
| At2g02010 | putative glutamate decarboxylase ; supported by cDNA: gi_13605709_gb_AF361836.1_AF361836 | 2.38282 |
| At2g15020 | hypothetical protein predicted by genscan and genefinder | 2.36741 |
| At4g24010 | putative protein cellulose synthase catalytic subunit (Ath-A), Arabidopsis thaliana; gb:AF027173 | 2.3657 |
| At1g21110 | O-methyltransferase, putative similar to GB:AAF28353 from [Fragaria x ananassa] | 2.36235 |
| At1g74210 | putative glycerophosphodiester phosphodiesterase similar to glycerophosphoryl diester phosphodiesterase GB:AAF12f49 from [Deinococcus radiodurans];supported by full-length cDNA: Ceres:24182. | 2.36153 |
| At4g24450 | putative protein hypothetical protein R1 - Solanum tuberosum,PIR2:T07050 | 2.35756 |
| At3g14280 | unknown protein | 2.35019 |
| At5g43850 | putative protein similar to unknown protein (pir\|\|T02918); supported by full-length cDNA: Ceres: 26596. | 2.34329 |
| At5g46230 | unknown protein ;supported by full-length cDNA: Ceres:15495. | 2.34193 |
| At5g43150 | unknown protein | 2.32071 |
| At4g25830 | Expressed protein ; supported by full-length cDNA: Ceres: 9546. | 2.31309 |
| At3g21890 | zinc finger protein, putative contains Pfam profile: PF01760 CONSTANS family zinc finger;supported by full-length cDNA: Ceres:258241. | 2.30875 |
| At3g14720 | putative MAP kinase similar to GB:AAD52659 from [Oryza sativa] | 2.30329 |
| At5g06510 | transcription factor-like protein ;supported by full-length cDNA: Ceres:114015. | 2.29987 |
| At2g43570 | endochitinase isolog | 2.29898 |
| At5g48570 | peptidylprolyl isomerase | 2.28192 |
| At2g43510 | putative trypsin inhibitor ; supported by cDNA: gi_15292710_gb_AY050789.1_ | 2.26799 |
| At1g04990 | zinc finger protein 2, putative similar to GB:AAD33770 from [Arabidopsis thaliana]; supported by cDNA: gi_15081720_gb_AY048253.1_ | 2.26385 |
| At5g53200 | putative protein contains similarity to MYB family transcription factor | 2.25552 |
| At1g62960 | 1-aminocyclopropane-1-carboxylate synthase, putative similar to GB:U35779 from [Triticum aestivum] (Plant Mol. Biol. 31 (5), 1009-1020 (1996)); supported by cDNA: gi_15451221_gb_AY054691.1_ | 2.23557 |
| At2g29370 | putative tropinone reductase | 2.23246 |
| At4g08870 | putative arginase similar to arginases (Pfam: PF00491, Score=353.2, E=1.4e-119, N=1);supported by full-length cDNA: Ceres:33985. | 2.22635 |
| At4g35320 | putative protein predicted protein, Arabidopsis thaliana;supported by full-length cDNA: Ceres:4354. | 2.22615 |
| At3g10940 | unknown protein ;supported by full-length cDNA: Ceres:3056. | 2.22391 |
| At3g21560 | UDP-glucose:indole-3-acetate beta-D-glucosyltransferase, putative similar to UDP-glucose:indole-3-acetate beta-D-glucosyltransferase GB:AAB58497 | 2.22314 |
| At4g31870 | glutathione peroxidase - like protein glutathione peroxidase, Arabidopsis thaliana, PIR2:S71250 | 2.22112 |
| At2g36970 | putative glucosyltransferase ; supported by cDNA: gi_15809993_gb_AY054265.1_ | 2.21821 |
| At5g46240 | potassium channel protein KAT1 (pir\|\|S32816) ; supported by cDNA: gi_166773_gb_M86990.1_ATHKAT1 | 2.2171 |
| At3g57680 | carboxyl terminal protease - like protein carboxyl terminal protease, Nostoc punctiforme, AF022823 | 2.21638 |
| At2g38210 | similar to SOR1 from the fungus Cercospora nicotianae | 2.21119 |
| At2g16890 | putative glucosyltransferase ;supported by full-length cDNA: Ceres:153418. | 2.20922 |
| At4g33140 | hypothetical protein ; supported by cDNA: gi_15982924_gb_AY057570.1_ | 2.19315 |
| At2g45720 | unknown protein | 2.18984 |
| At1g13990 | unknown protein ;supported by full-length cDNA: Ceres:93312. | 2.1839 |
| At5g51720 | unknown protein ; supported by full-length cDNA: Ceres: 266744. | 2.1803 |
| At3g45970 | putative protein cim1 induced allergen, Glycine max, EMBL:U03860;supported by full-length cDNA: Ceres:27534. | 2.166 |
| At5g17600 | RING-H2 zinc finger protein-like RING-H2 zinc finger protein ATL4 - Arabidopsis thaliana, EMBL:AF132014 | 2.16582 |
| At1g22370 | UDP-glucose glucosyltransferase, putative similar to UDP-glucose glucosyltransferase GI:3928543 from [Arabidopsis thaliana]; supported by cDNA: gi_14532545_gb_AY039897.1_ | 2.16536 |
| At3g03480 | putative hypersensitivity-related gene similar to hypersensitivity-related gene GB:CAA64636 [Nicotiana tabacum];supported by full-length cDNA: Ceres:113502. | 2.16324 |
| At3g22550 | unknown protein | 2.16144 |
| At3g55605 | Expressed protein ; supported by full-length cDNA: Ceres: 250217. | 2.15708 |
| At2g17500 | unknown protein ;supported by full-length cDNA: Ceres:105072. | 2.15312 |
| At2g34850 | putative UDP-galactose-4-epimerase | 2.15218 |
| At3g44970 | cytochrome P450 - like protein cytochrome P450 d13695c, Arabidopsis thaliana, PIR:C71417 | 2.15151 |
| At5g03650 | 1,4-alpha-glucan branching enzyme protein soform SBE2.2 precursor | 2.14661 |
| At3g50910 | putative protein ; supported by cDNA: gi_14517539_gb_AY039605.1_ | 2.14653 |
| At3g13672 | seven in absentia-like protein similar to SINA1p GB:CAB40577 from [Vitis vinifera]; supported by cDNA: gi_16323493_gb_AY059135.1_ | 2.13692 |
| At2g40130 | hypothetical protein predicted by genscan and genefinder; supported by cDNA: gi_15450730_gb_AY053407.1_ | 2.12773 |
| At1g17870 | hypothetical protein predicted by genscan+ | 2.11449 |
| At3g29590 | Anthocyanin 5-aromatic acyltransferase, putative similar to Anthocyanin 5-aromatic acyltransferase GB:BAA74428 from [Gentiana triflora] | 2.10838 |
| At5g13800 | putative protein similar to unknown protein (pir\|\|S77419); supported by cDNA: gi_15912216_gb_AY056386.1_ | 2.10548 |
| At3g52710 | hypothetical protein predicted protein, Arabidopsis thaliana | 2.09959 |
| At1g16730 | hypothetical protein predicted by genemark.hmm | 2.0971 |
| At5g45840 | receptor protein kinase-like protein predicted by genemarkHMM and genscan+ | 2.09696 |
| At4g34710 | arginine decarboxylase SPE2 ; supported by cDNA: gi_14517491_gb_AY039581.1_ | 2.09513 |
| At4g01480 | putative inorganic phosphatase ; supported by cDNA: gi_15450871_gb_AY054516.1_ | 2.09361 |
| At2g21970 | unknown protein ;supported by full-length cDNA: Ceres:10672. | 2.09344 |
| At4g28270 | putative protein Arabidopsis thaliana RMA1 mRNA, PID:d1029538 | 2.09025 |
| At2g30540 | putative glutaredoxin ;supported by full-length cDNA: Ceres:39560. | 2.08825 |
| At1g54100 | aldehyde dehydrogenase homolog, putative similar to aldehyde dehydrogenase homolog GI:913941 from [Brassica napus]; supported by cDNA: gi_14190390_gb_AF378873.1_AF378873 | 2.08345 |
| At5g17760 | BCS1 - like protein h-bcs1, Homo sapiens, EMBL:AF026849; supported by cDNA: gi_15810570_gb_AY056324.1_ | 2.05916 |
| At3g24470 | hypothetical protein predicted by genscan+ | 2.05463 |
| At3g03470 | putative cytochrome P450 similar to cytochrome P450 89A2 GB:Q42602 [Arabidopsis thaliana]; supported by cDNA: gi_15983413_gb_AF424581.1_AF424581 | 2.04923 |
| At4g17695 | Expressed protein ; supported by cDNA: gi_15723596_gb_AY048690.1_ | 2.047 |
| At1g06430 | FtsH protease, putative similar to zinc dependent protease GI:7650138 from [Arabidopsis thaliana] | 2.04666 |
| At5g18130 | putative protein similar to unknown protein (gb\|AAF00631.1);supported by full-length cDNA: Ceres:10299. | 2.03757 |
| At1g10970 | ZIP4, a putative zinc transporter per suggestion by Dr. Natasha M. Grotz (PNAS, Vol 95., 7220-7224) | 2.03753 |
| At4g18800 | ras-like GTP-binding protein strong homology to GTP-binding protein ric2, Oryza sativa, S38741 | 2.02628 |
| At1g27980 | sphingosine-1-phosphate lyase, putative similar to sphingosine-1-phosphate lyase [Homo sapiens] GI:4160532; supported by cDNA: gi_13430507_gb_AF360166.1_AF360166 | 2.02095 |
| At1g37130 | nitrate reductase, putative similar to nitrate reductase GI:540486 from [Brassica napus]; supported by cDNA: gi_14194132_gb_AF367272.1_AF367272 | 2.01667 |
| At1g64810 | unknown protein | 2.01514 |
| At4g00670 | hypothetical protein | 2.01465 |
| At2g34720 | putative CCAAT-binding transcription factor subunit | 2.01183 |
| At5g58690 | phosphoinositide-specific phospholipase - like protein phosphoinositide-specific phospholipase C PLC1, Solanum tuberosum, PIR:T07421 | 2.00653 |
| At1g30500 | transcription factor, putative similar to transcription factor GB:CAA74050 GI:2398525 from [Arabidopsis thaliana] | 2.00536 |
| At1g73700 | putative integral membrane protein contains Pfam profile: PF01554 uncharacterized membrane protein family;supported by full-length cDNA: Ceres:125771. | 2.00353 |
| At5g24870 | RING finger-like protein RING-H2 finger protein RHG1a - Arabidopsis thaliana, EMBL:AF079183 | 2.0008 |

***DOWNREGULATION***

| **Transcript ID** | **Target Description** | **Fold Change** |
| --- | --- | --- |
| At5g46690 | putative protein contains similarity to bHLH DNA-binding protein;supported by full-length cDNA: Ceres:35890. | -2.0009 |
| At3g23080 | unknown protein C-term similar to phosphatidylcholine transfer protein GB:AAF08345 [Homo sapiens]; supported by cDNA: gi_15810256_gb_AY056167.1_ | -2.00483 |
| At5g06860 | polygalacturonase inhibiting protein 1; PGIP1 (gb\|AAF69827.1) ;supported by full-length cDNA: Ceres:5344. | -2.00633 |
| At1g72450 | unknown protein ;supported by full-length cDNA: Ceres:40573. | -2.00698 |
| At4g04630 | putative protein A. thaliana hypothetical protein F1N20.70, GenBank accession number AL022140;supported by full-length cDNA: Ceres:118778. | -2.0086 |
| At1g52000 | myrosinase binding protein, putative similar to myrosinase binding protein GI:1711295 from [Brassica napus] | -2.00863 |
| At4g16000 | hypothetical protein ;supported by full-length cDNA: Ceres:9209. | -2.01354 |
| At5g08280 | hydroxymethylbilane synthase ;supported by full-length cDNA: Ceres:3051. | -2.01444 |
| At3g10610 | putative 40S ribosomal protein S17 similar to 40S ribosomal protein S17 GB:AAD50774 [Lycopersicon esculentum];supported by full-length cDNA: Ceres:4775. | -2.01528 |
| At1g27460 | hypothetical protein predicted by genscan+; supported by cDNA: gi_16226497_gb_AF428415.1_AF428415 | -2.01776 |
| At4g35770 | senescence-associated protein sen1 identical to senescence-associated protein GI:1046270 from [Arabidopsis thaliana] ;supported by full-length cDNA: Ceres:13699. | -2.01887 |
| At3g47800 | aldose 1-epimerase - like protein aldose 1-epimerase, Nicotiana tabacum, PIR:T01933;supported by full-length cDNA: Ceres:119313. | -2.0195 |
| At2g46330 | unknown protein ; supported by cDNA: gi_15294169_gb_AF410276.1_AF410276 | -2.02725 |
| At3g23030 | auxin-inducible gene (IAA2) identical to auxin-inducible gene (IAA2) GB:AF027157 [Arabidopsis thaliana] (Plant Physiol. 115, 1730 (1997)) | -2.03541 |
| At2g01890 | putative purple acid phosphatase contains metallo-phosphoesterase motif (PS50185) | -2.03574 |
| At1g22710 | putative sucrose transport protein, SUC2 strong similarity to GB:S38196 sucrose transport protein SUC2 from [Arabidopsis thaliana];supported by full-length cDNA: Ceres:7894. | -2.03747 |
| At1g29450 | auxin-induced protein, putative similar to SP:P33083 from [Glycine max];supported by full-length cDNA: Ceres:29931. | -2.03894 |
| At1g15000 | serine carboxypeptidase precursor, putative similar to GB:AAD42963 from [Matricaria chamomilla] | -2.03908 |
| At5g57660 | CONSTANS-like B-box zinc finger protein-like ;supported by full-length cDNA: Ceres:6639. | -2.04033 |
| At1g23020 | putative superoxide-generating NADPH oxidase flavocytochrome highly similar to GB:CAA70769, FRO1 and GB:CAA70770, FRO2 from [Arabidopsis thaliana] | -2.04099 |
| At1g69040 | unknown protein | -2.04237 |
| At1g11260 | glucose transporter almost identical to glucose transporter GB:P23586 from [Arabidopsis thaliana]; supported by full-length cDNA: Ceres: 33727. | -2.04247 |
| At5g59480 | putative ripening-related protein - like putative ripening-related protein Vitis vinifera EMBL:VVI237988;supported by full-length cDNA: Ceres:99078. | -2.04315 |
| At5g13740 | transporter-like protein | -2.04674 |
| At2g24570 | putative WRKY-type DNA binding protein ; supported by cDNA: gi_15991743_gb_AF425836.1_AF425836 | -2.04861 |
| At2g25735 | Expressed protein ; supported by full-length cDNA: Ceres: 7152. | -2.05344 |
| At5g53450 | putative protein similar to unknown protein (sp\|P29618);supported by full-length cDNA: Ceres:95459. | -2.05664 |
| At5g49360 | xylosidase | -2.05905 |
| At3g06980 | putative DEAD/DEAH box helicase contains Pfam profile: PF00270 DEAD/DEAH box helicase; supported by cDNA: gi_15081734_gb_AY048260.1_ | -2.05941 |
| At3g16460 | putative lectin contains Pfam profile: PF01419 jacalin-like lectin domain; similar to jasmonate inducible protein GB:Y11483 (Brassica napus), myrosinase binding protein GB:BAA84545 (Arabidopsis thaliana); supported by cDNA: gi_14334869_gb_AY035108.1_ | -2.06203 |
| At1g27210 | unknown protein supported by cDNA: Ceres: 103034, cDNA may not be full-length in this case.; supported by cDNA: gi_15982865_gb_AY057540.1_ | -2.06247 |
| At2g26710 | putative cytochrome P450 | -2.06689 |
| At3g49940 | putative protein hypothetical protein - Arabidopsis thaliana, EMBL:CAB38293;supported by full-length cDNA: Ceres:17840. | -2.07118 |
| At2g36050 | hypothetical protein predicted by genefinder; supported by cDNA: gi_14994264_gb_AY044326.1_ | -2.07246 |
| At1g03220 | unknown protein Strong similarity to gb\|D14550 extracellular dermal glycoprotein (EDGP) precursor from Daucus carota. ESTs gb\|H37281, gb\|T44167, gb\|T21813, gb\|N38437, gb\|Z26470, gb\|R65072, gb\|N76373, gb\|F15470, gb\|Z35182, gb\|H76373, gb\|Z34678 and gb\|Z35387 come from this | -2.08366 |
| At1g20190 | expansin S2 precursor, putative similar to GB:U30460 from [Cucumis sativus];supported by full-length cDNA: Ceres:11011. | -2.08821 |
| At3g54720 | Peptidase - like protein ileal peptidase, Rattus norvegicus, EMBL:AF009921;supported by full-length cDNA: Ceres:103193. | -2.09264 |
| At1g30820 | CTP synthase-like protein similar to ctp synthase (sp\|P17812\|PYRG_HUMAN); similar to ESTs gb\|AA660762, gb\|AA220982, dbj\|AU008137, gb\|AI054783, and gb\|AA100804 | -2.09562 |
| At5g08330 | putative protein auxin-induced basic helix-loop-helix transcription factor, Gossypium hirsutum, EMBL:AF165924; supported by cDNA: gi_15724267_gb_AF412074.1_AF412074 | -2.099 |
| At2g30930 | unknown protein ;supported by full-length cDNA: Ceres:125631. | -2.09972 |
| At4g19410 | putative pectinacetylesterase protein pectinacetylesterase precursor, Vigna radiata, PIR2:S68805;supported by full-length cDNA: Ceres:34674. | -2.10231 |
| At4g38850 | small auxin up RNA (SAUR-AC1) ;supported by full-length cDNA: Ceres:14973. | -2.10257 |
| At2g34930 | putative disease resistance protein | -2.10295 |
| At4g34760 | putative auxin-regulated protein auxin-induced protein X15, Glycine max, PIR2:JQ1097;supported by full-length cDNA: Ceres:10510. | -2.10356 |
| At3g14990 | 4-methyl-5(b-hydroxyethyl)-thiazole monophosphate biosynthesis protein, putative similar to ThiJ GB:AAA82704 [Escherichia coli]; likely encodes two ThiJ subunits within a single polypeptide; supported by cDNA: gi_14517477_gb_AY039574.1_ | -2.10839 |
| At4g12970 | putative protein | -2.1099 |
| At5g05860 | glucuronosyl transferase-like protein ; supported by cDNA: gi_15010631_gb_AY045617.1_ | -2.11082 |
| At5g65730 | xyloglucan endo-transglycosylase-like protein ;supported by full-length cDNA: Ceres:12301. | -2.11478 |
| At5g61590 | ethylene responsive element binding factor - like ethylene responsive element binding factor 5, Arabidopsis thaliana, SWISSPROT:ERF5_ARATH;supported by full-length cDNA: Ceres:19893. | -2.11537 |
| At4g12420 | putative pollen-specific protein pollen-specific protein precursor - common tobacco, PIR2:S22495 | -2.11775 |
| At5g19120 | conglutin gamma - like protein conglutin gamma precursor, Lupinus angustifolius, PIR:S21426; supported by cDNA: gi_15010797_gb_AY045700.1_ | -2.122 |
| At2g39870 | unknown protein ; supported by cDNA: gi_13877742_gb_AF370134.1_AF370134 | -2.14068 |
| At2g27830 | unknown protein ;supported by full-length cDNA: Ceres:33700. | -2.14251 |
| At4g36670 | sugar transporter like protein | -2.14419 |
| At1g50010 | tubulin alpha-2/alpha-4 chain, putative identical to tubulin alpha-2/alpha-4 chain GB:P29510 from [Arabidopsis thaliana]; supported by cDNA: gi_15294277_gb_AF410330.1_AF410330 | -2.14991 |
| At3g56060 | mandelonitrile lyase-like protein ADHESION OF CALYX EDGES (ACE) - Arabidopsis thaliana; supported by cDNA: gi_15982754_gb_AY057484.1_ | -2.16127 |
| At4g38860 | putative auxin-induced protein auxin-induced protein 10A, Glycine max., PIR2:JQ1099 | -2.17001 |
| At4g16515 | Expressed protein ; supported by full-length cDNA: Ceres: 6580. | -2.17146 |
| At1g44350 | IAA-amino acid hydrolase, putative similar to IAA-amino acid hydrolase GI:3421384 from [Arabidopsis thaliana] | -2.17562 |
| At5g13140 | putative protein ;supported by full-length cDNA: Ceres:14064. | -2.18 |
| At3g02560 | putative 40S ribosomal protein similar to ribosomal protein S7 GB:AAD26256 from [Secale cereale];supported by full-length cDNA: Ceres:30349. | -2.18249 |
| At5g13100 | putative protein ;supported by full-length cDNA: Ceres:39563. | -2.18499 |
| At1g75960 | AMP-binding protein, putative contains Pfam profile: PF00501 AMP-binding enzyme | -2.18606 |
| At3g54400 | nucleoid DNA-binding - like protein nucleoid DNA-binding protein cnd41, chloroplast, common tobacco, PIR:T01996; supported by full-length cDNA: Ceres: 8987. | -2.19115 |
| At5g16030 | putative protein with poly glutamic acid stretch hypothetical protein F16B3.13 - Arabidopsis thaliana, EMBL:AC021640; supported by full-length cDNA: Ceres: 29745. | -2.19123 |
| At3g15630 | unknown protein ; supported by cDNA: gi_15529233_gb_AY052241.1_ | -2.19137 |
| At2g15090 | putative fatty acid elongase | -2.20064 |
| At3g05800 | hypothetical protein predicted by genscan+;supported by full-length cDNA: Ceres:21672. | -2.20206 |
| At4g22190 | hypothetical protein ;supported by full-length cDNA: Ceres:6848. | -2.20659 |
| At2g05380 | unknown protein ; supported by cDNA: gi_15146251_gb_AY049267.1_ | -2.21498 |
| At2g45470 | unknown protein ;supported by full-length cDNA: Ceres:7709. | -2.22441 |
| At3g17770 | dihydroxyacetone kinase, putative similar to dihydroxyacetone kinase GB:AAC83220 [Schizosaccharomyces pombe] (Biochim. Biophys. Acta 1442 (2-3), 361-368 (1998)) | -2.2255 |
| At1g02450 | unknown protein | -2.22793 |
| At1g19670 | unknown protein contains similarity to chlorophyllase GI:7415999 from [Chenopodium album]; supported by full-length cDNA: Ceres: 31589. | -2.22977 |
| At5g62920 | response regulator 6 (ARR6) ; supported by cDNA: gi_3953600_dbj_AB008489.1_AB008489 | -2.23295 |
| At2g32100 | hypothetical protein predicted by genscan | -2.23395 |
| At5g23820 | putative protein similar to unknown protein (pir\|\|T00970); supported by cDNA: gi_15215722_gb_AY050390.1_ | -2.23646 |
| At4g17460 | homeobox-leucine zipper protein HAT1 (hd-zip protein 1) ;supported by full-length cDNA: Ceres:34167. | -2.23702 |
| At1g52200 | unknown protein ; supported by full-length cDNA: Ceres: 16614. | -2.23738 |
| At4g17245 | Expressed protein ; supported by cDNA: gi_15809953_gb_AY054245.1_ | -2.23799 |
| At5g63180 | pectate lyase ; supported by cDNA: gi_16648839_gb_AY058197.1_ | -2.23824 |
| At2g22980 | putative serine carboxypeptidase I ; supported by cDNA: gi_15294269_gb_AF410326.1_AF410326 | -2.24094 |
| At3g18290 | zinc finger protein, putative contains Pfam profiles: PF00097 Zinc finger, C3HC4 type (RING finger) (2 copies) | -2.2418 |
| At4g00950 | putative protein | -2.24686 |
| At3g02170 | unknown protein ;supported by full-length cDNA: Ceres:22225. | -2.25409 |
| At5g62360 | DC1.2 homologue - like protein DC1.2 homologue, Nicotiana tabacum, EMBL:AB009888 | -2.26416 |
| At3g50970 | dehydrin Xero2 ; supported by cDNA: gi_15809983_gb_AY054260.1_ | -2.2646 |
| At4g31290 | predicted protein cation transport protein ChaC, Escherichia coli, PIR2:G64868;supported by full-length cDNA: Ceres:39740. | -2.27782 |
| At2g46710 | putative rac GTPase activating protein | -2.28982 |
| At4g25470 | DRE CRT-binding protein DREB1C involved in low-temperature-responsive gene expression00; supported by cDNA: gi_3738227_dbj_AB007789.1_AB007789 | -2.29023 |
| At1g09390 | putative lipase Similar to nodulins and lipase; location of EST E6C2T7 , gb\|AA042309. similar to nodulins gi\|3328240, gi\|2129854 and others and lipase, gi\|2129636 | -2.2923 |
| At5g46330 | receptor protein kinase | -2.29599 |
| At2g34810 | putative berberine bridge enzyme | -2.29897 |
| At2g33330 | unknown protein ;supported by full-length cDNA: Ceres:35273. | -2.3027 |
| At1g77210 | sugar carrier protein, putative similar to sugar carrier protein GI:169735 from [Ricinus communis] | -2.30361 |
| At5g44020 | vegetative storage protein-like ;supported by full-length cDNA: Ceres:27372. | -2.30656 |
| At3g60290 | SRG1 - like protein SRG1 protein, Arabidopsis thaliana, PIR:S44261 | -2.31862 |
| At2g42870 | unknown protein ;supported by full-length cDNA: Ceres:102453. | -2.32227 |
| At3g10020 | unknown protein predicted by genefinder, multiple est matches;supported by full-length cDNA: Ceres:7073. | -2.33922 |
| At4g36540 | putative protein ; supported by full-length cDNA: Ceres: 123997. | -2.34897 |
| At1g09350 | putative galactinol synthase similar to GB:AAD26116 from [Brassica napus]; supported by cDNA: gi_13899102_gb_AF370546.1_AF370546 | -2.35202 |
| At3g60130 | beta-glucosidase-like protein several beta-glucosidases - different species; supported by cDNA: gi_15028300_gb_AY045953.1_ | -2.35442 |
| At5g47220 | ethylene responsive element binding factor 2 (ATERF2) (sp\|O80338) ;supported by full-length cDNA: Ceres:3012. | -2.35777 |
| At5g08030 | glycerophosphodiester phosphodiesterase - like protein glycerophosphodiester phosphodiesterase, Borrelia hermsii, EMBL:BH40762 | -2.36868 |
| At1g18810 | unknown protein ;supported by full-length cDNA: Ceres:151637. | -2.37053 |
| At5g62720 | putative protein predicted proteins, Arabidopsis thaliana; supported by full-length cDNA: Ceres: 38273. | -2.38845 |
| At4g27450 | putative protein stem-specific protein - Nicotiana tabacum,PID:g20037;supported by full-length cDNA: Ceres:35207. | -2.39309 |
| At1g73480 | lysophospholipase homolog, putative similar to lysophospholipase homolog GI:2801536 from [Oryza sativa]; supported by cDNA: gi_15028212_gb_AY045929.1_ | -2.39425 |
| At5g49100 | unknown protein ;supported by full-length cDNA: Ceres:116332. | -2.39559 |
| At2g39400 | putative phospholipase ; supported by cDNA: gi_14532651_gb_AY039950.1_ | -2.39633 |
| At3g46880 | hypothetical protein | -2.40278 |
| At5g44260 | putative protein similar to unknown protein (gb AAD10689.1); supported by cDNA: gi_14334449_gb_AY034916.1_ | -2.40483 |
| At1g17860 | lemir (miraculin), putative similar to lemir (miraculin) GI:2654440 from [Lycopersicon esculentum]; supported by cDNA: gi_12083239_gb_AF332416.1_AF332416 | -2.42005 |
| At5g22920 | PGPD14 protein ;supported by full-length cDNA: Ceres:41666. | -2.43132 |
| At5g12050 | putative serine rich protein predicted proteins, Arabidopsis thaliana;supported by full-length cDNA: Ceres:36958. | -2.44767 |
| At4g24780 | putative pectate lyase pectate lyase, Musa acuminata, PATX:E209876;supported by full-length cDNA: Ceres:36681. | -2.45511 |
| At1g68190 | putative zinc finger protein similar to zinc finger protein GB:BAA33206 from [Oryza sativa] | -2.45872 |
| At1g14890 | unknown protein similar to pectinesterase GB:X85216 GI:732912 [Phaseolus vulgaris];supported by full-length cDNA: Ceres:33355. | -2.46794 |
| At3g01490 | putative protein kinase similar to ATMRK1, an Arabidopsis protein kinase related to mammal mixed-lineage kinases and Raf protein kinases GB:BAA22079 [Arabidopsis thaliana]; supported by cDNA: gi_16323086_gb_AY057647.1_ | -2.48251 |
| At5g01015 | Expressed protein ; supported by full-length cDNA: Ceres: 32396. | -2.48365 |
| At3g05880 | low temperature and salt responsive protein LTI6A identical to low temperature and salt responsive protein LTI6A GB:AAC97512 from [Arabidopsis thaliana] | -2.48882 |
| At1g33600 | hypothetical protein similar to putative disease resistance protein GB:AAC14512 GI:2739389 from [Arabidopsis thaliana] | -2.49649 |
| At1g05575 | Expressed protein ; supported by full-length cDNA: Ceres: 27081. | -2.5098 |
| At5g44530 | subtilisin-like serine protease contains similarity to meiotic serine proteinase TMP GI:6468325 from [Lycopersicon esculentum] | -2.51052 |
| At3g62930 | glutaredoxin -like protein glutaredoxin, castor bean, PIR:S54825 | -2.52223 |
| At1g64380 | AP2-containing DNA-binding protein contains Pfam profile: PF00847 AP2 domain | -2.5309 |
| At3g14440 | 9-cis-epoxycarotenoid dioxygenase, putative similar to 9-cis-epoxycarotenoid dioxygenase GB:AAF26356 [Phaseolus vulgaris]; supported by cDNA: gi_15810432_gb_AY056255.1_ | -2.53535 |
| At2g40400 | unknown protein ; supported by cDNA: gi_15294187_gb_AF410285.1_AF410285 | -2.54409 |
| At1g26800 | hypothetical protein predicted by genscan+;supported by full-length cDNA: Ceres:250127. | -2.54827 |
| At5g25190 | ethylene-responsive element - like protein ethylene-responsive element binding protein homolog, Stylosanthes hamata, EMBL:U91857; supported by cDNA: gi_15010715_gb_AY045659.1_ | -2.55124 |
| At5g62280 | putative protein predicted proteins, Arabidopsis thaliana | -2.55257 |
| At1g67330 | unknown protein similar to hypothetical protein GI:9665091 from [Arabidopsis thaliana] | -2.55327 |
| At1g19180 | unknown protein ;supported by full-length cDNA: Ceres:38751. | -2.55991 |
| At1g01620 | plasma membrane intrinsic protein 1c, putative similar to plasma membrane intrinsic protein 1c GI:472875 from [Arabidopsis thaliana] | -2.56255 |
| At3g28310 | unknown protein | -2.56288 |
| At3g23510 | cyclopropane-fatty-acyl-phospholipid synthase, putative similar to cyclopropane-fatty-acyl-phospholipid synthase GB:P30010 [Escherichia coli] | -2.56416 |
| At5g50740 | putative protein contains similarity to ATFP3 | -2.56815 |
| At3g16240 | delta tonoplast integral protein (delta-TIP) identical to delta tonoplast integral protein (delta-TIP) GB:U39485 [Arabidopsis thaliana] (Plant Cell 8 (4), 587-599 (1996)); supported by full-length cDNA: Ceres: 19689. | -2.5744 |
| At5g05600 | leucoanthocyanidin dioxygenase-like protein ;supported by full-length cDNA: Ceres:13012. | -2.58254 |
| At2g28630 | putative fatty acid elongase ;supported by full-length cDNA: Ceres:115769. | -2.59422 |
| At5g44680 | putative protein contains similarity to DNA-3-methyladenine glycosylase I;supported by full-length cDNA: Ceres:29551. | -2.59616 |
| At5g40450 | putative protein microtubule-associated protein homolog, Drosophila melanogaster, EMBL:T13564 | -2.60257 |
| At2g05790 | putative beta-1,3-glucanase | -2.61385 |
| At5g05690 | cytochrome P450 90A1 (sp\|Q42569) ; supported by full-length cDNA: Ceres: 36334. | -2.62452 |
| At1g75250 | myb-related protein similar to GI:7981380 from [Lycopersicon esculentum] | -2.62676 |
| At3g07010 | putative pectate lyase similar to pectate lyase 2 GB:AAF19196 [Musa acuminata]; supported by full-length cDNA: Ceres: 124816. | -2.63342 |
| At2g22330 | putative cytochrome P450 | -2.6514 |
| At1g33340 | unknown protein | -2.66313 |
| At3g16800 | protein phosphatase, putative similar to protein phosphatase-2C GB:AAC36699 from [Mesembryanthemum crystallinum];supported by full-length cDNA: Ceres:6305. | -2.6775 |
| At3g16670 | unknown protein | -2.67893 |
| At3g28860 | P-glycoprotein, putative similar to P-glycoprotein (PGY1) GB:AAA59575 [Homo sapiens] | -2.68331 |
| At1g13260 | DNA-binding protein RAV1 identical to RAV1 GI:3868857 from [Arabidopsis thaliana]; supported by cDNA: gi_3868856_dbj_AB013886.1_AB013886 | -2.68564 |
| At5g02160 | putative protein ; supported by cDNA: gi_13358223_gb_AF325034.2_AF325034 | -2.68967 |
| At1g29460 | auxin-induced protein, putative similar to SP:P33083 from [Glycine max];supported by full-length cDNA: Ceres:147801. | -2.69381 |
| At1g29440 | auxin-induced protein, putative similar to SP:P33083 from [Glycine max] | -2.69524 |
| At5g21170 | AKIN beta1 ; supported by full-length cDNA: Ceres: 41668. | -2.7034 |
| At4g14550 | IAA7 like protein | -2.70452 |
| At2g16660 | nodulin-like protein | -2.71096 |
| At1g70290 | trehalose-6-phosphate synthase, putative similar to trehalose-6-phosphate synthase GI:297792 from [Kluyveromyces lactis] | -2.71447 |
| At4g01460 | putative bHLH DNA-binding protein | -2.71482 |
| At1g32090 | hypothetical protein similar to hypothetical protein GB:CAB77775 GI:7269775 from [Arabidopsis thaliana] | -2.73639 |
| At5g20250 | seed imbitition protein-like seed imbitition protein Sip1 - Hordeum vulgare, EMBL:M77475 | -2.7389 |
| At1g29430 | auxin-induced protein, putative similar to SP:P33083 from [Glycine max];supported by full-length cDNA: Ceres:4119. | -2.79146 |
| At2g20670 | unknown protein ;supported by full-length cDNA: Ceres:34827. | -2.82411 |
| At5g15350 | putative protein many predicted proteins, Arabidopsis thaliana; supported by cDNA: gi_15983504_gb_AF424627.1_AF424627 | -2.83192 |
| At4g16985 | Expressed protein ; supported by full-length cDNA: Ceres: 30087. | -2.8445 |
| At5g67420 | putative protein similar to unknown protein (emb\|CAB62102.1);supported by full-length cDNA: Ceres:40250. | -2.87769 |
| At1g32540 | zinc-finger protein, putative similar to GI:5262161 from [Arabidopsis thaliana]; supported by cDNA: gi_16323142_gb_AY057675.1_ | -2.88966 |
| At5g48540 | 33 kDa secretory protein-like ; supported by cDNA: gi_15292980_gb_AY050924.1_ | -2.90334 |
| At1g09750 | hypothetical protein predicted by genscan;supported by full-length cDNA: Ceres:6295. | -2.93409 |
| At3g07350 | unknown protein similar to hypothetical protein GB:AAC17612 [Arabidopsis thaliana];supported by full-length cDNA: Ceres:251012. | -2.98117 |
| At3g08030 | unknown protein ;supported by full-length cDNA: Ceres:27471. | -3.03549 |
| At4g24570 | putative mitochondrial uncoupling protein mitochondrial uncoupling protein, Arabidopsis thaliana (thale cress), PATX:E1316826;supported by full-length cDNA: Ceres:119476. | -3.13209 |
| At1g80440 | unknown protein contains two Kelch motifs; supported by full-length cDNA: Ceres: 32885. | -3.17057 |
| At4g39640 | putative gamma-glutamyltransferase gamma-glutamyltransferase, Arabidopsis thaliana, PIR2:S58286 | -3.18106 |
| At1g49500 | unknown protein ;supported by full-length cDNA: Ceres:33047. | -3.18276 |
| At2g06850 | putative endoxyloglucan glycosyltransferase identical to GB:D16454;supported by full-length cDNA: Ceres:15276. | -3.19107 |
| At2g28950 | expansin AtEx6 identical to GB U30480; supported by full-length cDNA: Ceres: 17914. | -3.19299 |
| At5g18060 | auxin-induced protein-like | -3.20743 |
| At2g25900 | putative CCCH-type zinc finger protein identical to GB:U81238;supported by full-length cDNA: Ceres:39893. | -3.21182 |
| At1g52190 | peptide transporter, putative similar to peptide transporter PTR2-B SP:P46032 [Arabidopsis thaliana (Mouse-ear cress)]; supported by full-length cDNA: Ceres: 113723 and gi:16648852 | -3.21269 |
| At1g52400 | beta-glucosidase, putative identical to GI:6651430 from [Arabidopsis thaliana]; supported by cDNA: gi_14532461_gb_AY039855.1_ | -3.22384 |
| At3g10720 | putative pectinesterase contains similarity to pectinesterase GB:AAB57671 [Citrus sinensis] | -3.23842 |
| At2g18700 | putative trehalose-6-phosphate synthase | -3.25088 |
| At5g66590 | putative protein contains similarity to pathogenesis-related protein;supported by full-length cDNA: Ceres:2152. | -3.25704 |
| At3g14210 | myrosinase-associated protein, putative similar to GB:CAA71238 from [Brassica napus], conatains Pfam profile:PF00657 Lipase/Acylhydrolase with GDSL-like motif; supported by cDNA: gi_15450434_gb_AY052318.1_ | -3.26925 |
| At1g66940 | unknown protein identical to unknown protein GI:9755444 from (Arabidopsis thaliana); supported by full-length cDNA: Ceres: 110066. | -3.32291 |
| At5g06870 | polygalacturonase inhibiting protein ;supported by full-length cDNA: Ceres:35527. | -3.33635 |
| At3g06070 | unknown protein predicted by genscan+;supported by full-length cDNA: Ceres:153279. | -3.36639 |
| At3g48100 | response reactor 2 (ATRR2) ; supported by cDNA: gi_3273197_dbj_AB010916.1_AB010916 | -3.36739 |
| At3g58120 | putative protein basic leucine zipper transcription activator shoot-forming PKSF1 - Paulownia kawakamii, EMBL:AF046934;supported by full-length cDNA: Ceres:34553. | -3.37176 |
| At4g23820 | putative polygalacturonase polygalacturonase (EC 3.2.1.15) - avocado, EMBL:X66426 | -3.38148 |
| At2g44740 | putative PREG1-like negative regulator | -3.461 |
| At4g04840 | putative protein similar to transcriptional regulator | -3.58891 |
| At2g18300 | hypothetical protein predicted by genscan; supported by cDNA: gi_15724317_gb_AF412099.1_AF412099 | -3.63434 |
| At3g01960 | hypothetical protein predicted by genscan | -3.68761 |
| At1g29500 | auxin-induced protein, putative similar to SP:P33083 from [Glycine max] | -3.76221 |
| At3g05730 | unknown protein | -3.77395 |
| At5g24420 | 6-phosphogluconolactonase-like protein ;supported by full-length cDNA: Ceres:13806. | -3.78472 |
| At4g25490 | transcriptional activator CBF1 CRT CRE binding factor 1 involved in low-temperature-responsive gene expression00; supported by cDNA: gi_1899057_gb_U77378.1_ATU77378 | -3.81589 |
| At4g31820 | putative protein various predicted proteins | -3.99555 |
| At3g16470 | putative lectin contains Pfam profile: PF01419 jacalin-like lectin domain; similar to jasmonate inducible protein GB:Y11483 (Brassica napus), myrosinase binding protein GB:BAA84545 (Arabidopsis thaliana);supported by full-length cDNA: Ceres:30003. | -4.02763 |
| At1g25230 | hypothetical protein similar to putative purple acid phosphatase precursor GI:7331195 from [Glycine max] | -4.10903 |
| At5g03350 | putative protein | -4.18933 |
| At1g18620 | unknown protein | -4.22254 |
| At3g30180 | cytochrome P450 homolog, putative similar to cytochrome P450 homolog GB:U54770 GI:1421740 from [Lycopersicon esculentum];supported by full-length cDNA: Ceres:11278. | -4.26674 |
| At1g66690 | unknown protein | -4.32911 |
| At1g69530 | expansin (At-EXP1) identical to expansin (At-EXP1) [Arabidopsis thaliana] GI:1041702;supported by full-length cDNA: Ceres:255048. | -4.47566 |
| At5g18600 | glutaredoxin -like protein glutaredoxin, castor bean, PIR:S54825;supported by full-length cDNA: Ceres:92720. | -4.51867 |
| At5g40890 | anion channel protein (gb\|AAC05742.1) ; supported by cDNA: gi_1619955_gb_U72151.1_ATU72151 | -4.53159 |
| At5g50335 | Expressed protein ; supported by full-length cDNA: Ceres: 15527. | -4.5345 |
| At1g68560 | alpha-xylosidase precursor identical to alpha-xylosidase precursor GB:AAD05539 GI:4163997 from [Arabidopsis thaliana]; supported by cDNA: gi_15982750_gb_AY057482.1_ | -4.61918 |
| At2g10940 | unknown protein ;supported by full-length cDNA: Ceres:32647. | -4.87166 |
| At4g32280 | Expressed protein ; supported by cDNA: gi_14190492_gb_AF380646.1_AF380646 | -4.87855 |
| At2g26530 | AR781, similar to yeast pheromone receptor identical to GB:D88743, corrected a frameshift found in the original record (at 69530 bp), sequence submitted has been verified from 10 sequence electropherograms. The translation now starts from an upstream ATG.;supported by full-length cDNA: Ceres:33680 | -4.96412 |
| At5g39860 | putative protein putative DNA-binding protein - Arabidopsis thaliana, EMBL:AC011765;supported by full-length cDNA: Ceres:4734. | -5.00311 |
| At3g28320 | At14a-like protein similar to At14a protein GB:AAD26355 GI:4589123 [Arabidopsis thaliana] | -5.04887 |
| At1g29510 | auxin-induced protein, putative similar to SP:P33083 from [Glycine max];supported by full-length cDNA: Ceres:9311. | -5.1217 |
| At1g52040 | myrosinase-binding protein homolog, putative almost identical to myrosinase-binding protein homolog GI:2997767 from [Arabidopsis thaliana]; supported by cDNA: gi_13937207_gb_AF372958.1_AF372958 | -5.1346 |
| At1g52410 | myosin-like protein contains Pfam profile: PF00658 Poly-adenylate binding protein, unique domain.; supported by cDNA: gi_15081708_gb_AY048247.1_ | -5.22018 |
| At3g22120 | unknown protein similar to cell wall-plasma membrane linker protein GB:CAA64425 from [Brassica napus] | -5.71623 |
| At3g15450 | unknown protein very similar to unknown protein GB:AAC39468 from [Arabidopsis thaliana]; supported by cDNA: gi_14335087_gb_AY037223.1_ | -6.09753 |
| At5g25460 | putative protein hypothetical protein - Ricinus communis, EMBL:Z81012;supported by full-length cDNA: Ceres:1351. | -6.43426 |
| At2g20750 | beta-expansin ;supported by full-length cDNA: Ceres:109135. | -6.51693 |
| At3g62950 | glutaredoxin -like protein glutaredoxin, castor bean, PIR:S54825 | -7.23372 |
| At3g28220 | unknown protein | -8.17452 |
| At5g02760 | protein phosphatase - like protein protein phosphatase 2C homolog, Mesembryanthemum crystallinum, EMBL:AF097667 | -8.86006 |
| At2g40610 | putative expansin ;supported by full-length cDNA: Ceres:27553. | -8.99263 |
| At5g48490 | putative protein similar to unknown protein (pir\|\|S72530);supported by full-length cDNA: Ceres:32925. | -9.95054 |
| At1g74670 | GAST1-like protein similar to GAST1 protein precursor GB:P27057 [Lycopersicon esculentum] (induced by gibberellins, inhibited by ABA Plant J 1992 Mar;2(2):153-9) | -22.851 |

***gpt2*HL/WTLL**

***UPREGULATION***

| **Transcript ID** | **Target Description** | **Fold Change** |
| --- | --- | --- |
| At5g24780 | vegetative storage protein Vsp1 ;supported by full-length cDNA: Ceres:32606. | 37.8436674 |
| At5g59310 | nonspecific lipid-transfer protein precursor - like nonspecific lipid-transfer protein precursor, Brassica napus, EMBL:AF101038;supported by full-length cDNA: Ceres:43057. | 21.8942481 |
| At5g59320 | nonspecific lipid-transfer protein precursor - like nonspecific lipid-transfer protein precursor, Brassica napus, EMBL:AF101038;supported by full-length cDNA: Ceres:7828. | 21.722677 |
| At3g15650 | putative lysophospholipase similar to lysophospholipase GB:AAD52700 [Schistosoma japonicum] | 14.2674935 |
| At1g56650 | anthocyanin2, putative similar to anthocyanin2 (An2) GI:7673088 from [Petunia integrifolia]; supported by cDNA: gi_3941507_gb_AF062908.1_AF062908 | 13.848617 |
| At5g58770 | dehydrodolichyl diphosphate - like protein dehydrodolichyl diphosphate, Arabidopsis thaliana, EMBL:ATH277136 | 11.2562159 |
| At2g40100 | putative chlorophyll a/b binding protein ; supported by full-length cDNA: Ceres: 6454. | 7.76647529 |
| At4g16590 | cellulose synthase like protein | 7.64231646 |
| At1g32900 | starch synthase, putative similar to starch synthase GI:21613 from [Solanum tuberosum];supported by full-length cDNA: Ceres:7714. | 7.16109398 |
| At4g23990 | cellulose synthase catalytic subunit - like protein cellulose synthase catalytic subunit (Ath-A), Arabidopsis thaliana, gb:AF027173 | 7.02783344 |
| At1g64780 | ammonium transporter, puitative similar to ammonium transporter GI:5880357 from [Arabidopsis thaliana]; supported by cDNA: gi_4324713_gb_AF110771.1_AF110771 | 6.41171367 |
| At2g21590 | putative ADP-glucose pyrophosphorylase large subunit | 6.20245774 |
| At3g08860 | putative aminotransferase similar to beta-alanine-pyruvate aminotransferase GB:BAA19549 [Rattus norvegicus], alanine-glyoxylate aminotransferase GB:Q64565 [Rattus norvegicus]; Pfam HMM hit: Aminotransferases class-III pyridoxal-phosphate | 6.17945669 |
| At4g39210 | glucose-1-phosphate adenylyltransferase (APL3) ; supported by cDNA: gi_16648984_gb_AY059862.1_ | 5.90108402 |
| At5g19470 | putative protein thiamin pyrophosphokinase, Schizosaccharomyces pombe, PIR:S52350 | 5.11668706 |
| At1g52770 | putative non-phototropic hypocotyl similar to non-phototropic hypocotyl 3 GB:AAF05914 from [Arabidopsis thaliana] | 5.07849141 |
| At1g73040 | jacalin, putative similar to jacalin GI:289164 from [Artocarpus heterophyllus] | 4.97242634 |
| At2g43570 | endochitinase isolog | 4.93842158 |
| At1g14250 | hypothetical protein | 4.80701065 |
| At2g27420 | cysteine proteinase contains similarity to cysteine protease SPCP1 GI:13491750 from [Ipomoea batatas] | 4.7476053 |
| At5g11930 | glutaredoxin - like protein glutaredoxin, R.communis, EMBL:RCGLREDXN | 4.58362812 |
| At2g37760 | putative alcohol dehydrogenase ; supported by cDNA: gi_16604706_gb_AY059798.1_ | 4.48059398 |
| At1g21400 | branched-chain alpha keto-acid dehydrogenase, putative similar to branched-chain alpha keto-acid dehydrogenase GB:AAC69851 GI:3822223 from [Arabidopsis thaliana] | 4.32664874 |
| At1g62710 | beta-VPE nearly identical to beta-VPE GB:BAA09615 GI:1805364 [Arabidopsis thaliana]; supported by cDNA: gi_14194096_gb_AF367254.1_AF367254 | 4.16524108 |
| At1g67360 | stress related protein, putative similar to stress related protein GI:5802955 from [Vitis riparia];supported by full-length cDNA: Ceres:14043. | 3.97832186 |
| At1g80130 | unknown protein ;supported by full-length cDNA: Ceres:35675. | 3.87664869 |
| At1g02850 | beta-glucosidase, putative similar to beta-glucosidase GI:5030906 from [Polygonum tinctorium]; supported by cDNA: gi_15146265_gb_AY049274.1_ | 3.83203304 |
| At2g43510 | putative trypsin inhibitor ; supported by cDNA: gi_15292710_gb_AY050789.1_ | 3.82147608 |
| At5g54060 | flavonol 3-O-glucosyltransferase-like | 3.63163699 |
| At1g77920 | transcription factor, putative similar to transcription factor GI:304113 from [Arabidopsis thaliana]; supported by cDNA: gi_16209662_gb_AY057596.1_ | 3.38055472 |
| At2g43580 | putative endochitinase | 3.36993321 |
| At2g25625 | Expressed protein ; supported by full-length cDNA: Ceres: 465. | 3.36965292 |
| At3g49580 | putative protein ;supported by full-length cDNA: Ceres:26235. | 3.36244344 |
| At3g12580 | heat shock protein 70 identical to heat shock protein 70 GB:CAA05547 GI:3962377 [Arabidopsis thaliana]; supported by cDNA: gi_15809831_gb_AY054183.1_ | 3.33769008 |
| At3g17790 | acid phosphatase type 5 identical to GB:CAB63938 from [Arabidopsis thaliana];supported by full-length cDNA: Ceres:113666. | 3.29616851 |
| At5g37980 | quinone oxidoreductase -like protein probable quinone oxidoreductase P1, Arabidopsis thaliana;supported by full-length cDNA: Ceres:116237. | 3.25891111 |
| At1g57590 | pectinacetylesterase precursor, putative similar to pectinacetylesterase precursor GI:1431629 from [Vigna radiata] | 3.22104764 |
| At4g08870 | putative arginase similar to arginases (Pfam: PF00491, Score=353.2, E=1.4e-119, N=1);supported by full-length cDNA: Ceres:33985. | 3.12845424 |
| At3g10450 | putative glucose acyltransferase similar to glucose acyltransferase GB:AAD01263 [Solanum berthaultii]; also similar to serine carboxypeptidase I GB:P37890 [Oryza sativa]; supported by full-length cDNA: Ceres: 94163. | 3.04029223 |
| At4g37150 | hydroxynitrile lyase like protein | 3.00201253 |
| At5g53200 | putative protein contains similarity to MYB family transcription factor | 3.0010347 |
| At5g65870 | putative protein similar to unknown protein (emb CAB66916.1);supported by full-length cDNA: Ceres:9323. | 2.91367507 |
| At1g76530 | unknown protein | 2.88076105 |
| At3g21560 | UDP-glucose:indole-3-acetate beta-D-glucosyltransferase, putative similar to UDP-glucose:indole-3-acetate beta-D-glucosyltransferase GB:AAB58497 | 2.87778737 |
| At5g64550 | putative protein strong similarity to unknown protein (emb\|CAB89363.1) | 2.86481107 |
| At5g53420 | putative protein similar to unknown protein (pir\|\|T02891);supported by full-length cDNA: Ceres:112574. | 2.84569294 |
| At4g24010 | putative protein cellulose synthase catalytic subunit (Ath-A), Arabidopsis thaliana; gb:AF027173 | 2.78334549 |
| At1g21460 | hypothetical protein contains similarity to MTN3 (nodule development protein) GB:Y08726 GI:1619601 from [Medicago truncatula];supported by full-length cDNA: Ceres:6411. | 2.77937404 |
| At5g52390 | photoassimilate-responsive protein PAR-like protein ;supported by full-length cDNA: Ceres:17872. | 2.77662049 |
| At4g34590 | bZIP transcription factor ATB2 | 2.75404093 |
| At5g24120 | sigma-like factor (emb CAA77213.1) | 2.74971101 |
| At1g11700 | unknown protein ESTs gb\|R65381 and gb\|T44635 come from this gene | 2.72487491 |
| At4g26950 | putative protein other Arabidopsis hypothetical proteins | 2.70948752 |
| At2g46680 | homeodomain transcription factor (ATHB-7) identical to SP:P46897; supported by cDNA: gi_15027938_gb_AY045826.1_ | 2.70699083 |
| At3g58070 | zinc finger-like protein several zinc finger proteins - Arabidopsis thaliana | 2.70455268 |
| At2g18230 | putative inorganic pyrophosphatase ;supported by full-length cDNA: Ceres:8068. | 2.69067847 |
| At4g20170 | putative protein gene F4P9.34 chromosome II BAC F4P9, Arabidopsis thaliana | 2.68242903 |
| At5g25140 | cytochrome P450-like protein CYTOCHROME P450 71B1 - Thlaspi arvense, EMBL:L24438 | 2.65995161 |
| At5g03210 | putative protein | 2.63510418 |
| At2g15020 | hypothetical protein predicted by genscan and genefinder | 2.63367988 |
| At3g55605 | Expressed protein ; supported by full-length cDNA: Ceres: 250217. | 2.62247678 |
| At1g22160 | unknown protein ;supported by full-length cDNA: Ceres:23788. | 2.61318633 |
| At4g17770 | trehalose-6-phosphate synthase like protein ;supported by full-length cDNA: Ceres:95947. | 2.59814149 |
| At3g47420 | putative protein sn-glycerol-3-phosphate permease - Haemophilus influenzae,PID:g3603157; supported by cDNA: gi_13430515_gb_AF360170.1_AF360170 | 2.59450623 |
| At2g36885 | Expressed protein ; supported by full-length cDNA: Ceres: 29157. | 2.591846 |
| At4g34710 | arginine decarboxylase SPE2 ; supported by cDNA: gi_14517491_gb_AY039581.1_ | 2.58811191 |
| At4g25830 | Expressed protein ; supported by full-length cDNA: Ceres: 9546. | 2.53859456 |
| At1g10970 | ZIP4, a putative zinc transporter per suggestion by Dr. Natasha M. Grotz (PNAS, Vol 95., 7220-7224) | 2.53430472 |
| At2g32210 | unknown protein ;supported by full-length cDNA: Ceres:31665. | 2.52721779 |
| At1g62960 | 1-aminocyclopropane-1-carboxylate synthase, putative similar to GB:U35779 from [Triticum aestivum] (Plant Mol. Biol. 31 (5), 1009-1020 (1996)); supported by cDNA: gi_15451221_gb_AY054691.1_ | 2.50736148 |
| At3g26290 | cytochrome P450, putative contains Pfam profile: PF00067 cytochrome P450 | 2.50520732 |
| At1g07300 | hypothetical protein predicted by genemark.hmm | 2.50366233 |
| At4g16690 | cyanohydrin lyase like protein ;supported by full-length cDNA: Ceres:5546. | 2.50343674 |
| At4g17880 | bHLH protein - like bHLH protein, Arabidopsis thaliana, PATCHX:E255557 | 2.49695531 |
| At3g22840 | early light-induced protein identical to early light-induced protein GB:AAB88391 from [Arabidopsis thaliana];supported by full-length cDNA: Ceres:14490. | 2.47747414 |
| At2g40460 | putative PTR2 family peptide transporter | 2.47522556 |
| At3g22550 | unknown protein | 2.47111131 |
| At5g46240 | potassium channel protein KAT1 (pir\|\|S32816) ; supported by cDNA: gi_166773_gb_M86990.1_ATHKAT1 | 2.46416694 |
| At3g14720 | putative MAP kinase similar to GB:AAD52659 from [Oryza sativa] | 2.46314234 |
| At4g23260 | putative protein receptor protein kinase, Ipomoea trifida | 2.41236799 |
| At2g34850 | putative UDP-galactose-4-epimerase | 2.41057948 |
| At5g48570 | peptidylprolyl isomerase | 2.40745695 |
| At1g74210 | putative glycerophosphodiester phosphodiesterase similar to glycerophosphoryl diester phosphodiesterase GB:AAF12f49 from [Deinococcus radiodurans];supported by full-length cDNA: Ceres:24182. | 2.39756512 |
| At5g10380 | putative protein various predicted proteins, Arabidopsis thaliana | 2.37838126 |
| At4g24450 | putative protein hypothetical protein R1 - Solanum tuberosum,PIR2:T07050 | 2.37815047 |
| At5g24870 | RING finger-like protein RING-H2 finger protein RHG1a - Arabidopsis thaliana, EMBL:AF079183 | 2.37679916 |
| At4g17670 | hypothetical protein ;supported by full-length cDNA: Ceres:6709. | 2.36016873 |
| At1g37130 | nitrate reductase, putative similar to nitrate reductase GI:540486 from [Brassica napus]; supported by cDNA: gi_14194132_gb_AF367272.1_AF367272 | 2.35446618 |
| At5g43850 | putative protein similar to unknown protein (pir\|\|T02918); supported by full-length cDNA: Ceres: 26596. | 2.34941244 |
| At4g23320 | serine/threonine kinase - like protein serine/threonine kinase, Brassica oleracea | 2.34913561 |
| At3g13110 | serine acetyltransferase (Sat-1) identical to serine acetyltransferase (Sat-1) GB:U22964 [Arabidopsis thaliana] (Plant Mol. Biol. 30 (5), 1041-1049 (1996)); supported by cDNA: gi_1184047_gb_U22964.1_ATU22964 | 2.3456699 |
| At1g33110 | unknown protein | 2.33353968 |
| At4g15530 | pyruvate,orthophosphate dikinase | 2.3159113 |
| At1g55210 | unknown protein | 2.2993403 |
| At5g17600 | RING-H2 zinc finger protein-like RING-H2 zinc finger protein ATL4 - Arabidopsis thaliana, EMBL:AF132014 | 2.28000976 |
| At3g44970 | cytochrome P450 - like protein cytochrome P450 d13695c, Arabidopsis thaliana, PIR:C71417 | 2.25058492 |
| At3g23920 | beta-amylase, putative similar to beta-amylase GB:CAB58423 [Arabidopsis thaliana]; supported by cDNA: gi_14194172_gb_AF367293.1_AF367293 | 2.2443225 |
| At3g52180 | putative protein ;supported by full-length cDNA: Ceres:114734. | 2.2382947 |
| At4g33905 | Expressed protein ; supported by cDNA: gi_13358221_gb_AF325032.2_AF325032 | 2.23424903 |
| At1g28570 | lipase, putative contains Pfam profile: PF00657 Lipase/Acylhydrolase with GDSL-like motif | 2.22530024 |
| At2g38210 | similar to SOR1 from the fungus Cercospora nicotianae | 2.22391246 |
| At4g15490 | indole-3-acetate beta-glucosyltransferase like protein ;supported by full-length cDNA: Ceres:35383. | 2.21744758 |
| At2g21320 | putative CONSTANS-like B-box zinc finger protein ; supported by cDNA: gi_16974588_gb_AY060570.1_ | 2.20728053 |
| At1g77070 | MADS-box protein (MADS2), putative similar to MADS-box protein (MADS2) GI:886400 from [Oryza sativa] | 2.19935425 |
| At5g43150 | unknown protein | 2.19907986 |
| At2g30540 | putative glutaredoxin ;supported by full-length cDNA: Ceres:39560. | 2.19350805 |
| At5g54130 | putative protein similar to unknown protein (gb\|AAC24386.1) | 2.17979128 |
| At1g04220 | putative beta-ketoacyl-CoA synthase Strong similarity to beta-keto-Coa synthase gb\|U37088 from Simmondsia chinensis | 2.17143685 |
| At5g41400 | RING zinc finger protein-like ;supported by full-length cDNA: Ceres:207148. | 2.16576994 |
| At4g31870 | glutathione peroxidase - like protein glutathione peroxidase, Arabidopsis thaliana, PIR2:S71250 | 2.1632344 |
| At5g14760 | L-aspartate oxidase -like protein L-aspartate oxidase, Pseudomonas aeruginosa, PIR:T46863; supported by cDNA: gi_15010649_gb_AY045626.1_ | 2.16130099 |
| At1g78510 | prenyltransferase, putative similar to prenyltransferase GI:1651651 from [Synechocystis sp.] | 2.14931956 |
| At5g41070 | putative protein similar to unknown protein (gb\|AAD20688.1) | 2.1457619 |
| At1g02270 | unknown protein ; supported by cDNA: gi_15450672_gb_AY052704.1_ | 2.13513901 |
| At2g41120 | hypothetical protein predicted by genefinder | 2.13479864 |
| At2g32290 | putative beta-amylase | 2.1208315 |
| At1g09500 | putative cinnamyl alcohol dehydrogenase similar to cinnamyl alcohol dehydrogenase, gi\|1143445; supported by cDNA: gi_15983385_gb_AF424567.1_AF424567 | 2.12030234 |
| At4g16790 | glycoprotein homolog ;supported by full-length cDNA: Ceres:255542. | 2.10697033 |
| At4g21990 | PRH26 protein ;supported by full-length cDNA: Ceres:36866. | 2.10489753 |
| At5g47330 | palmitoyl-protein thioesterase precursor-like | 2.10323492 |
| At5g17760 | BCS1 - like protein h-bcs1, Homo sapiens, EMBL:AF026849; supported by cDNA: gi_15810570_gb_AY056324.1_ | 2.09420112 |
| At4g33140 | hypothetical protein ; supported by cDNA: gi_15982924_gb_AY057570.1_ | 2.08650735 |
| At3g50910 | putative protein ; supported by cDNA: gi_14517539_gb_AY039605.1_ | 2.08503269 |
| At4g38020 | hypothetical protein | 2.08247619 |
| At4g35320 | putative protein predicted protein, Arabidopsis thaliana;supported by full-length cDNA: Ceres:4354. | 2.08012467 |
| At5g02260 | expansin precursor - like protein expansin precursor Exp4, Lycopersicon esculentum, EMBL:AF059488; supported by full-length cDNA: Ceres: 21205. | 2.07836638 |
| At1g80830 | metal ion transporter identical to GB:AAD54417 GI:5853313 from [Arabidopsis thaliana];supported by full-length cDNA: Ceres:250751. | 2.071506 |
| At5g09590 | heat shock protein 70 (Hsc70-5) ; supported by cDNA: gi_6746589_gb_AF217458.1_AF217458 | 2.06196522 |
| At1g61930 | hypothetical protein similar to hypothetical protein GI:2894571 from [Arabidopsis thaliana];supported by full-length cDNA: Ceres:30230. | 2.05605663 |
| At3g50280 | anthranilate N-hydroxycinnamoyl/benzoyltransferase - like protein anthranilate N-hydroxycinnamoyl/benzoyltransferase, Dianthus caryophyllus, PIR:T10717 | 2.05317984 |
| At3g16910 | AMP-binding protein, putative contains Pfam profile: PF00501 AMP-binding enzyme; similar to acyl-CoA synthetase GB:CAB54055 [Pseudomonas putida] | 2.0521981 |
| At5g01300 | putative protein several hypothetical proteins - different bacteria | 2.04267545 |
| At1g78290 | serine-threonine protein kinase, putative similar to serine-threonine protein kinase (TaPK3) GI:2055372 from [Triticum aestivum];supported by full-length cDNA: Ceres:98655. | 2.0405386 |
| At1g15410 | hypothetical protein Is a member of the PF\|01177 Aspartate-glutamate racemase family. EST gb\|T43554 comes from this gene | 2.03133756 |
| At5g39670 | calcium-binding protein - like cbp1 calcium-binding protein, Lotus japonicus, EMBL:LJA251808; supported by cDNA: gi_16648829_gb_AY058192.1_ | 2.02893128 |
| At3g52720 | carbonic anhydrase (CAH1) ; supported by cDNA: gi_15450772_gb_AY054466.1_ | 2.02404308 |
| At1g11840 | lactoylglutathione lyase-like protein Similar to protein gb\|Z74962 from Brassica oleracea which is similar to bacterial YRN1 and HEAHIO proteins. ESTs gb\|T21954, gb\|T04283, gb\|Z37609, gb\|N37366, gb\|R90704, gb\|F15500 and gb\|F14353 come from this gene;supported by full-length cDNA: Ceres:39107. | 2.01502816 |
| At5g64650 | 50S ribosomal protein L17 ;supported by full-length cDNA: Ceres:2806. | 2.00722174 |
| At1g78020 | unknown protein ;supported by full-length cDNA: Ceres:6082. | 2.00510808 |
| At3g10525 | Expressed protein ; supported by full-length cDNA: Ceres: 2153. | 2.00144227 |
| At1g53280 | hypothetical protein contains Pfam profile: PF01965 ThiJ/PfpI family;supported by full-length cDNA: Ceres:101735. | 2.00013863 |

***DOWNREGULATION***

| **Transcript ID** | **Target Description** | **Fold Change** |
| --- | --- | --- |
| At4g00400 | putative protein | -2.0025385 |
| At3g08030 | unknown protein ;supported by full-length cDNA: Ceres:27471. | -2.0026079 |
| At5g49170 | putative protein similar to unknown protein (gb\|AAF63814.1);supported by full-length cDNA: Ceres:40718. | -2.0042882 |
| At3g56480 | putative protein hypothetical protein At2g26770 - Arabidopsis thaliana, EMBL:AC003105 | -2.0056919 |
| At1g18870 | isochorismate synthase, putative similar to isochorismate synthase GI:3348077 from [Arabidopsis thaliana] | -2.0076113 |
| At1g49230 | RING-H2 finger protein RHA3a, putative similar to RING-H2 finger protein RHA3a GI:3790573 from [Arabidopsis thaliana] | -2.0132691 |
| At1g69890 | hypothetical protein similar to GB:AAB61488 [Arabidopsis thaliana];supported by full-length cDNA: Ceres:34864. | -2.0269212 |
| At1g48330 | hypothetical protein similar to hypothetical protein GI:9294146 from [Arabidopsis thaliana] | -2.027413 |
| At5g05860 | glucuronosyl transferase-like protein ; supported by cDNA: gi_15010631_gb_AY045617.1_ | -2.0441343 |
| At4g11360 | RING-H2 finger protein RHA1b ;supported by full-length cDNA: Ceres:37097. | -2.0497103 |
| At1g20190 | expansin S2 precursor, putative similar to GB:U30460 from [Cucumis sativus];supported by full-length cDNA: Ceres:11011. | -2.0500655 |
| At3g49790 | putative protein predicted protein, Arabidopsis thaliana | -2.0529379 |
| At5g62920 | response regulator 6 (ARR6) ; supported by cDNA: gi_3953600_dbj_AB008489.1_AB008489 | -2.0546889 |
| At2g42530 | cold-regulated protein cor15b precursor ;supported by full-length cDNA: Ceres:19221. | -2.0592229 |
| At1g22710 | putative sucrose transport protein, SUC2 strong similarity to GB:S38196 sucrose transport protein SUC2 from [Arabidopsis thaliana];supported by full-length cDNA: Ceres:7894. | -2.0603651 |
| At1g53830 | pectin methylesterase (PMEU1), putative similar to pectin methylesterase (PMEU1) GI:1222551 from [Lycopersicon esculentum]; supported by cDNA: gi_13605622_gb_AF361637.1_AF361637 | -2.0615794 |
| At4g30690 | putative protein translation initiation factor, IF3 - Listeria monocytogenes; supported by cDNA: gi_14596120_gb_AY042848.1_ | -2.0627515 |
| At3g14440 | 9-cis-epoxycarotenoid dioxygenase, putative similar to 9-cis-epoxycarotenoid dioxygenase GB:AAF26356 [Phaseolus vulgaris]; supported by cDNA: gi_15810432_gb_AY056255.1_ | -2.0632233 |
| At4g38690 | putative protein phospholipase C (EC 3.1.4.3) precursor,phosphatidylinositol-specific - Listeria monocytogenes, PIR2:A37204 | -2.0638527 |
| At5g14920 | putative protein predicted protein, Arabidopsis thaliana;supported by full-length cDNA: Ceres:32599. | -2.0696976 |
| At4g23400 | water channel - like protein plasma membrane intrinsic protein 1c, Arabidopsis thaliana, PIR2:S44083;supported by full-length cDNA: Ceres:3982. | -2.0708026 |
| At4g05070 | coded for by A. thaliana cDNA T44741 | -2.0711901 |
| At3g03990 | unknown protein similar to putative protein GB:CAB38214 [Arabidopsis thaliana];supported by full-length cDNA: Ceres:6052. | -2.0810765 |
| At3g23080 | unknown protein C-term similar to phosphatidylcholine transfer protein GB:AAF08345 [Homo sapiens]; supported by cDNA: gi_15810256_gb_AY056167.1_ | -2.0863627 |
| At3g07320 | putative glucan endo-1-3-beta-glucosidase similar to glucan endo-1-3-beta-glucosidase precursor GB:P52409 [Triticum aestivum];supported by full-length cDNA: Ceres:36049. | -2.0872306 |
| At2g28950 | expansin AtEx6 identical to GB U30480; supported by full-length cDNA: Ceres: 17914. | -2.0873608 |
| At4g32340 | putative protein predicted proteins, Arabidopsis thaliana;supported by full-length cDNA: Ceres:34819. | -2.0904304 |
| At5g59080 | putative protein | -2.0944189 |
| At3g02170 | unknown protein ;supported by full-length cDNA: Ceres:22225. | -2.1034973 |
| At3g09580 | putative oxidoreductase similar to phytoene desaturase GB:P28553 from [Glycine max];supported by full-length cDNA: Ceres:17350. | -2.106138 |
| At4g16515 | Expressed protein ; supported by full-length cDNA: Ceres: 6580. | -2.1120002 |
| At4g33666 | Expressed protein ; supported by full-length cDNA: Ceres: 38416. | -2.1179228 |
| At5g62720 | putative protein predicted proteins, Arabidopsis thaliana; supported by full-length cDNA: Ceres: 38273. | -2.1206992 |
| At1g01190 | cytochrome P450, putative similar to cytochrome P450 GB:L23209 GI:349717 from (Zea mays) | -2.1271621 |
| At5g56100 | unknown protein | -2.1273538 |
| At5g50740 | putative protein contains similarity to ATFP3 | -2.1275455 |
| At5g25280 | serine-rich protein ;supported by full-length cDNA: Ceres:99323. | -2.129803 |
| At5g19120 | conglutin gamma - like protein conglutin gamma precursor, Lupinus angustifolius, PIR:S21426; supported by cDNA: gi_15010797_gb_AY045700.1_ | -2.1414381 |
| At2g26710 | putative cytochrome P450 | -2.1415272 |
| At4g19410 | putative pectinacetylesterase protein pectinacetylesterase precursor, Vigna radiata, PIR2:S68805;supported by full-length cDNA: Ceres:34674. | -2.1423883 |
| At3g14990 | 4-methyl-5(b-hydroxyethyl)-thiazole monophosphate biosynthesis protein, putative similar to ThiJ GB:AAA82704 [Escherichia coli]; likely encodes two ThiJ subunits within a single polypeptide; supported by cDNA: gi_14517477_gb_AY039574.1_ | -2.1451968 |
| At1g29450 | auxin-induced protein, putative similar to SP:P33083 from [Glycine max];supported by full-length cDNA: Ceres:29931. | -2.1642693 |
| At1g04820 | tubulin alpha-2/alpha-4 chain identical to SP:P29510 from [Arabidopsis thaliana]; supported by cDNA: gi_16648843_gb_AY058199.1_ | -2.1693608 |
| At1g08980 | unknown protein Contains similarity to Rhodococcus amidase (gb\|D16207). ESTs gb\|T20504,gb\|H36650,gb\|N97423,gb\|H36595 come from this gene; supported by cDNA: gi_14335037_gb_AY037198.1_ | -2.170203 |
| At1g71030 | putative transcription factor similar to myb-related transcription factor 24 GB:S71287;supported by full-length cDNA: Ceres:31592. | -2.1793532 |
| At5g11420 | putative protein predicted proteins in castor bean, Arabidopsis thaliana and alfalfa.; supported by full-length cDNA: Ceres: 25522. | -2.1840411 |
| At1g27210 | unknown protein supported by cDNA: Ceres: 103034, cDNA may not be full-length in this case.; supported by cDNA: gi_15982865_gb_AY057540.1_ | -2.1852525 |
| At4g04840 | putative protein similar to transcriptional regulator | -2.1900442 |
| At4g38850 | small auxin up RNA (SAUR-AC1) ;supported by full-length cDNA: Ceres:14973. | -2.1907578 |
| At5g49450 | putative protein contains similarity to bZIP transcription factor;supported by full-length cDNA: Ceres:43004. | -2.1966728 |
| At4g39640 | putative gamma-glutamyltransferase gamma-glutamyltransferase, Arabidopsis thaliana, PIR2:S58286 | -2.2098983 |
| At1g13700 | unknown protein similar to 6-phosphogluconolactonase-like protein GI:10177902 from [Arabidopsis thaliana] | -2.2188313 |
| At1g10020 | unknown protein Location of EST gb\|T41885 and gb\|AA395021 | -2.2219248 |
| At1g18330 | hypothetical protein similar to hypothetical protein GB:AAF25987 GI:6714291 from [Arabidopsis thaliana] | -2.2225255 |
| At4g17245 | Expressed protein ; supported by cDNA: gi_15809953_gb_AY054245.1_ | -2.2232189 |
| At1g04240 | putative auxin-induced protein AUX2-11 Match to Arabidopsis IAA3 (gb\|U18406). EST gb\|T04296 comes from this gene; supported by cDNA: gi_972910_gb_U18406.1_ATU18406 | -2.2277078 |
| At4g01460 | putative bHLH DNA-binding protein | -2.2382792 |
| At5g03130 | putative protein | -2.245365 |
| At1g73480 | lysophospholipase homolog, putative similar to lysophospholipase homolog GI:2801536 from [Oryza sativa]; supported by cDNA: gi_15028212_gb_AY045929.1_ | -2.2458943 |
| At1g70420 | unknown protein ; supported by cDNA: gi_15010575_gb_AY045589.1_ | -2.2461278 |
| At2g44130 | unknown protein ;supported by full-length cDNA: Ceres:6950. | -2.2487293 |
| At2g37950 | unknown protein | -2.2523328 |
| At4g04630 | putative protein A. thaliana hypothetical protein F1N20.70, GenBank accession number AL022140;supported by full-length cDNA: Ceres:118778. | -2.267386 |
| At2g22980 | putative serine carboxypeptidase I ; supported by cDNA: gi_15294269_gb_AF410326.1_AF410326 | -2.2774352 |
| At4g01130 | putative acetyltransferase similar to Digitalis lanata lanatoside 15'-O-acetyltransferase, GenBank accession number AJ011567 | -2.2810531 |
| At4g03110 | putative ribonucleoprotein similarity to ovarian RNA-binding protein and translational control factor (bruno)- Drosphila melanogaster,EMBL:DMU58976;supported by full-length cDNA: Ceres:114653. | -2.2832677 |
| At2g27830 | unknown protein ;supported by full-length cDNA: Ceres:33700. | -2.2833626 |
| At5g46690 | putative protein contains similarity to bHLH DNA-binding protein;supported by full-length cDNA: Ceres:35890. | -2.2869265 |
| At5g46330 | receptor protein kinase | -2.2907182 |
| At2g36050 | hypothetical protein predicted by genefinder; supported by cDNA: gi_14994264_gb_AY044326.1_ | -2.2932919 |
| At1g14890 | unknown protein similar to pectinesterase GB:X85216 GI:732912 [Phaseolus vulgaris];supported by full-length cDNA: Ceres:33355. | -2.2939437 |
| At2g46710 | putative rac GTPase activating protein | -2.3349635 |
| At4g32290 | putative protein predicted protein, Arabidopsis thaliana, PATCHX:G2252634 | -2.3403755 |
| At4g16985 | Expressed protein ; supported by full-length cDNA: Ceres: 30087. | -2.3453447 |
| At1g09750 | hypothetical protein predicted by genscan;supported by full-length cDNA: Ceres:6295. | -2.3460439 |
| At3g62930 | glutaredoxin -like protein glutaredoxin, castor bean, PIR:S54825 | -2.3611996 |
| At1g29430 | auxin-induced protein, putative similar to SP:P33083 from [Glycine max];supported by full-length cDNA: Ceres:4119. | -2.3742799 |
| At1g09390 | putative lipase Similar to nodulins and lipase; location of EST E6C2T7 , gb\|AA042309. similar to nodulins gi\|3328240, gi\|2129854 and others and lipase, gi\|2129636 | -2.3887568 |
| At2g01420 | putative auxin transport protein ; supported by cDNA: gi_7109714_gb_AF087016.1_AF087016 | -2.3913578 |
| At5g01015 | Expressed protein ; supported by full-length cDNA: Ceres: 32396. | -2.3960201 |
| At5g25190 | ethylene-responsive element - like protein ethylene-responsive element binding protein homolog, Stylosanthes hamata, EMBL:U91857; supported by cDNA: gi_15010715_gb_AY045659.1_ | -2.4122844 |
| At2g20670 | unknown protein ;supported by full-length cDNA: Ceres:34827. | -2.421448 |
| At5g24490 | putative protein contains similarity to ribosomal protein 30S subunit; supported by cDNA: gi_13877770_gb_AF370148.1_AF370148 | -2.4299051 |
| At1g32540 | zinc-finger protein, putative similar to GI:5262161 from [Arabidopsis thaliana]; supported by cDNA: gi_16323142_gb_AY057675.1_ | -2.4548077 |
| At5g44530 | subtilisin-like serine protease contains similarity to meiotic serine proteinase TMP GI:6468325 from [Lycopersicon esculentum] | -2.4860064 |
| At2g01890 | putative purple acid phosphatase contains metallo-phosphoesterase motif (PS50185) | -2.5082654 |
| At5g50450 | putative protein strong similarity to unknown protein (gb\|AAB95234.1) | -2.5593013 |
| At1g29460 | auxin-induced protein, putative similar to SP:P33083 from [Glycine max];supported by full-length cDNA: Ceres:147801. | -2.5720345 |
| At4g14550 | IAA7 like protein | -2.5764418 |
| At4g04330 | hypothetical protein ; supported by cDNA: gi_15027850_gb_AY045782.1_ | -2.5843474 |
| At4g24800 | putative protein apoptosis gene MA3, Suberites domuncula, Y15421; supported by cDNA: gi_17063162_gb_AY062102.1_ | -2.5919897 |
| At1g66690 | unknown protein | -2.5986098 |
| At2g42870 | unknown protein ;supported by full-length cDNA: Ceres:102453. | -2.6312713 |
| At4g27450 | putative protein stem-specific protein - Nicotiana tabacum,PID:g20037;supported by full-length cDNA: Ceres:35207. | -2.6320922 |
| At1g68050 | F-box protein FKF1/ADO3, AtFBX2a identical to FKF1 GI:6960305 and Adagio 3 GI:13487072 from [Arabidopsis thaliana] | -2.633114 |
| At2g28630 | putative fatty acid elongase ;supported by full-length cDNA: Ceres:115769. | -2.6481411 |
| At1g18810 | unknown protein ;supported by full-length cDNA: Ceres:151637. | -2.6706113 |
| At3g01490 | putative protein kinase similar to ATMRK1, an Arabidopsis protein kinase related to mammal mixed-lineage kinases and Raf protein kinases GB:BAA22079 [Arabidopsis thaliana]; supported by cDNA: gi_16323086_gb_AY057647.1_ | -2.6840285 |
| At5g16030 | putative protein with poly glutamic acid stretch hypothetical protein F16B3.13 - Arabidopsis thaliana, EMBL:AC021640; supported by full-length cDNA: Ceres: 29745. | -2.6995146 |
| At2g25200 | hypothetical protein predicted by grail | -2.7077415 |
| At1g30820 | CTP synthase-like protein similar to ctp synthase (sp\|P17812\|PYRG_HUMAN); similar to ESTs gb\|AA660762, gb\|AA220982, dbj\|AU008137, gb\|AI054783, and gb\|AA100804 | -2.7493298 |
| At5g65730 | xyloglucan endo-transglycosylase-like protein ;supported by full-length cDNA: Ceres:12301. | -2.762166 |
| At5g62280 | putative protein predicted proteins, Arabidopsis thaliana | -2.7767552 |
| At2g30930 | unknown protein ;supported by full-length cDNA: Ceres:125631. | -2.7970964 |
| At5g60680 | putative protein predicted proteins, Arabidopsis thaliana;supported by full-length cDNA: Ceres:16638. | -2.7992493 |
| At5g06870 | polygalacturonase inhibiting protein ;supported by full-length cDNA: Ceres:35527. | -2.7993076 |
| At1g50010 | tubulin alpha-2/alpha-4 chain, putative identical to tubulin alpha-2/alpha-4 chain GB:P29510 from [Arabidopsis thaliana]; supported by cDNA: gi_15294277_gb_AF410330.1_AF410330 | -2.8151465 |
| At1g01620 | plasma membrane intrinsic protein 1c, putative similar to plasma membrane intrinsic protein 1c GI:472875 from [Arabidopsis thaliana] | -2.8457916 |
| At1g69160 | hypothetical protein predicted by genemark.hmm; supported by cDNA: gi_16648778_gb_AY058166.1_ | -2.8495024 |
| At3g47800 | aldose 1-epimerase - like protein aldose 1-epimerase, Nicotiana tabacum, PIR:T01933;supported by full-length cDNA: Ceres:119313. | -2.8597519 |
| At3g15630 | unknown protein ; supported by cDNA: gi_15529233_gb_AY052241.1_ | -2.8630443 |
| At2g10940 | unknown protein ;supported by full-length cDNA: Ceres:32647. | -2.8818195 |
| At2g39400 | putative phospholipase ; supported by cDNA: gi_14532651_gb_AY039950.1_ | -2.9192343 |
| At1g49500 | unknown protein ;supported by full-length cDNA: Ceres:33047. | -2.9220888 |
| At3g28860 | P-glycoprotein, putative similar to P-glycoprotein (PGY1) GB:AAA59575 [Homo sapiens] | -2.9278873 |
| At1g32090 | hypothetical protein similar to hypothetical protein GB:CAB77775 GI:7269775 from [Arabidopsis thaliana] | -2.9312784 |
| At1g77960 | hypothetical protein | -2.9359349 |
| At1g68190 | putative zinc finger protein similar to zinc finger protein GB:BAA33206 from [Oryza sativa] | -2.9364845 |
| At5g44260 | putative protein similar to unknown protein (gb AAD10689.1); supported by cDNA: gi_14334449_gb_AY034916.1_ | -2.9420464 |
| At4g17460 | homeobox-leucine zipper protein HAT1 (hd-zip protein 1) ;supported by full-length cDNA: Ceres:34167. | -2.9544918 |
| At3g10020 | unknown protein predicted by genefinder, multiple est matches;supported by full-length cDNA: Ceres:7073. | -2.9717855 |
| At3g48100 | response reactor 2 (ATRR2) ; supported by cDNA: gi_3273197_dbj_AB010916.1_AB010916 | -2.9738255 |
| At2g16660 | nodulin-like protein | -2.9919167 |
| At1g55330 | unknown protein ; supported by full-length cDNA: Ceres: 38378. | -2.9973551 |
| At3g61060 | putative protein hypothetical proteins - Arabidopsis thaliana; supported by cDNA: gi_14334587_gb_AY034967.1_ | -3.0014508 |
| At2g15890 | unknown protein ;supported by full-length cDNA: Ceres:29905. | -3.0040316 |
| At5g21170 | AKIN beta1 ; supported by full-length cDNA: Ceres: 41668. | -3.0305087 |
| At1g29440 | auxin-induced protein, putative similar to SP:P33083 from [Glycine max] | -3.0502342 |
| At5g40450 | putative protein microtubule-associated protein homolog, Drosophila melanogaster, EMBL:T13564 | -3.1275219 |
| At5g02160 | putative protein ; supported by cDNA: gi_13358223_gb_AF325034.2_AF325034 | -3.1428426 |
| At1g70290 | trehalose-6-phosphate synthase, putative similar to trehalose-6-phosphate synthase GI:297792 from [Kluyveromyces lactis] | -3.1916667 |
| At4g33980 | putative protein ;supported by full-length cDNA: Ceres:8161. | -3.2210253 |
| At5g05690 | cytochrome P450 90A1 (sp\|Q42569) ; supported by full-length cDNA: Ceres: 36334. | -3.2413392 |
| At3g60130 | beta-glucosidase-like protein several beta-glucosidases - different species; supported by cDNA: gi_15028300_gb_AY045953.1_ | -3.2525247 |
| At3g05880 | low temperature and salt responsive protein LTI6A identical to low temperature and salt responsive protein LTI6A GB:AAC97512 from [Arabidopsis thaliana] | -3.2950492 |
| At5g12050 | putative serine rich protein predicted proteins, Arabidopsis thaliana;supported by full-length cDNA: Ceres:36958. | -3.3117413 |
| At3g07350 | unknown protein similar to hypothetical protein GB:AAC17612 [Arabidopsis thaliana];supported by full-length cDNA: Ceres:251012. | -3.3252896 |
| At3g16800 | protein phosphatase, putative similar to protein phosphatase-2C GB:AAC36699 from [Mesembryanthemum crystallinum];supported by full-length cDNA: Ceres:6305. | -3.3334821 |
| At3g28310 | unknown protein | -3.3494634 |
| At2g18300 | hypothetical protein predicted by genscan; supported by cDNA: gi_15724317_gb_AF412099.1_AF412099 | -3.3532266 |
| At3g46640 | putative protein several hypothetical proteins - Arabidopsis thaliana | -3.384869 |
| At5g44680 | putative protein contains similarity to DNA-3-methyladenine glycosylase I;supported by full-length cDNA: Ceres:29551. | -3.4140401 |
| At5g62360 | DC1.2 homologue - like protein DC1.2 homologue, Nicotiana tabacum, EMBL:AB009888 | -3.4183734 |
| At5g24470 | putative protein contains similarity to two-component response regulator protein; supported by cDNA: gi_10281005_dbj_AB046955.1_AB046955 | -3.4594915 |
| At1g80440 | unknown protein contains two Kelch motifs; supported by full-length cDNA: Ceres: 32885. | -3.613057 |
| At1g07050 | hypothetical protein contains similarity to photoperiod sensitivity quantitative trait locus (Hd1) GI:11094203 from [Oryza sativa];supported by full-length cDNA: Ceres:1955. | -3.614585 |
| At1g26800 | hypothetical protein predicted by genscan+;supported by full-length cDNA: Ceres:250127. | -3.6227117 |
| At1g72150 | cytosolic factor, putative similar to GI:807956 from [Saccharomyces cerevisiae]; supported by cDNA: gi_15028180_gb_AY045913.1_ | -3.6563141 |
| At1g18620 | unknown protein | -3.6599146 |
| At3g22120 | unknown protein similar to cell wall-plasma membrane linker protein GB:CAA64425 from [Brassica napus] | -3.7247407 |
| At3g07010 | putative pectate lyase similar to pectate lyase 2 GB:AAF19196 [Musa acuminata]; supported by full-length cDNA: Ceres: 124816. | -3.7290031 |
| At2g32100 | hypothetical protein predicted by genscan | -3.865808 |
| At3g01960 | hypothetical protein predicted by genscan | -3.8718955 |
| At3g30180 | cytochrome P450 homolog, putative similar to cytochrome P450 homolog GB:U54770 GI:1421740 from [Lycopersicon esculentum];supported by full-length cDNA: Ceres:11278. | -4.0002773 |
| At1g29500 | auxin-induced protein, putative similar to SP:P33083 from [Glycine max] | -4.003828 |
| At5g66590 | putative protein contains similarity to pathogenesis-related protein;supported by full-length cDNA: Ceres:2152. | -4.0507807 |
| At1g52190 | peptide transporter, putative similar to peptide transporter PTR2-B SP:P46032 [Arabidopsis thaliana (Mouse-ear cress)]; supported by full-length cDNA: Ceres: 113723 and gi:16648852 | -4.0999889 |
| At5g18060 | auxin-induced protein-like | -4.1052498 |
| At5g49360 | xylosidase | -4.1522405 |
| At5g18600 | glutaredoxin -like protein glutaredoxin, castor bean, PIR:S54825;supported by full-length cDNA: Ceres:92720. | -4.1960736 |
| At3g05800 | hypothetical protein predicted by genscan+;supported by full-length cDNA: Ceres:21672. | -4.2292739 |
| At5g22920 | PGPD14 protein ;supported by full-length cDNA: Ceres:41666. | -4.381546 |
| At1g68560 | alpha-xylosidase precursor identical to alpha-xylosidase precursor GB:AAD05539 GI:4163997 from [Arabidopsis thaliana]; supported by cDNA: gi_15982750_gb_AY057482.1_ | -4.3881414 |
| At1g25230 | hypothetical protein similar to putative purple acid phosphatase precursor GI:7331195 from [Glycine max] | -4.4078343 |
| At2g25900 | putative CCCH-type zinc finger protein identical to GB:U81238;supported by full-length cDNA: Ceres:39893. | -4.6294795 |
| At5g61590 | ethylene responsive element binding factor - like ethylene responsive element binding factor 5, Arabidopsis thaliana, SWISSPROT:ERF5_ARATH;supported by full-length cDNA: Ceres:19893. | -4.7224647 |
| At4g23820 | putative polygalacturonase polygalacturonase (EC 3.2.1.15) - avocado, EMBL:X66426 | -4.7739055 |
| At5g40890 | anion channel protein (gb\|AAC05742.1) ; supported by cDNA: gi_1619955_gb_U72151.1_ATU72151 | -4.9187782 |
| At4g31820 | putative protein various predicted proteins | -4.9661574 |
| At2g06850 | putative endoxyloglucan glycosyltransferase identical to GB:D16454;supported by full-length cDNA: Ceres:15276. | -5.0123251 |
| At3g50970 | dehydrin Xero2 ; supported by cDNA: gi_15809983_gb_AY054260.1_ | -5.0321328 |
| At4g32280 | Expressed protein ; supported by cDNA: gi_14190492_gb_AF380646.1_AF380646 | -5.2904851 |
| At5g20250 | seed imbitition protein-like seed imbitition protein Sip1 - Hordeum vulgare, EMBL:M77475 | -5.4411817 |
| At1g29510 | auxin-induced protein, putative similar to SP:P33083 from [Glycine max];supported by full-length cDNA: Ceres:9311. | -5.4698831 |
| At2g44740 | putative PREG1-like negative regulator | -5.4939737 |
| At3g28320 | At14a-like protein similar to At14a protein GB:AAD26355 GI:4589123 [Arabidopsis thaliana] | -5.8374974 |
| At5g39860 | putative protein putative DNA-binding protein - Arabidopsis thaliana, EMBL:AC011765;supported by full-length cDNA: Ceres:4734. | -6.0640431 |
| At2g20750 | beta-expansin ;supported by full-length cDNA: Ceres:109135. | -6.0834512 |
| At3g62950 | glutaredoxin -like protein glutaredoxin, castor bean, PIR:S54825 | -6.1797994 |
| At5g25460 | putative protein hypothetical protein - Ricinus communis, EMBL:Z81012;supported by full-length cDNA: Ceres:1351. | -6.2805896 |
| At1g09350 | putative galactinol synthase similar to GB:AAD26116 from [Brassica napus]; supported by cDNA: gi_13899102_gb_AF370546.1_AF370546 | -6.357502 |
| At2g18700 | putative trehalose-6-phosphate synthase | -6.6900176 |
| At2g33830 | putative auxin-regulated protein ;supported by full-length cDNA: Ceres:1711. | -7.8625103 |
| At5g02760 | protein phosphatase - like protein protein phosphatase 2C homolog, Mesembryanthemum crystallinum, EMBL:AF097667 | -11.230809 |
| At2g40610 | putative expansin ;supported by full-length cDNA: Ceres:27553. | -11.281758 |
| At5g48490 | putative protein similar to unknown protein (pir\|\|S72530);supported by full-length cDNA: Ceres:32925. | -11.566817 |
| At3g15450 | unknown protein very similar to unknown protein GB:AAC39468 from [Arabidopsis thaliana]; supported by cDNA: gi_14335087_gb_AY037223.1_ | -11.756608 |
| At1g74670 | GAST1-like protein similar to GAST1 protein precursor GB:P27057 [Lycopersicon esculentum] (induced by gibberellins, inhibited by ABA Plant J 1992 Mar;2(2):153-9) | -20.719395 |

***gpt2*LL/WTLL**

***UPREGULATION***

| **Transcript ID** | **Target Description** | **Fold Change** |
| --- | --- | --- |
| At1g61800 | glucose-6-phosphate/phosphate-translocator precursor, putative similar to glucose-6-phosphate/phosphate-translocator precursor GI:2997591 from [Pisum sativum]; supported by cDNA: gi_14596172_gb_AY042874.1_ | 4.54335 |
| At2g43620 | putative endochitinase | 3.28131 |
| At5g65080 | MADS box transcription factor-like protein | 2.5672 |
| At1g77070 | MADS-box protein (MADS2), putative similar to MADS-box protein (MADS2) GI:886400 from [Oryza sativa] | 2.54246 |
| At3g49920 | porin - like protein porin, Mesembryanthemum crystallinum, PIR:T12558 | 2.41689 |
| At2g02100 | protease inhibitor II identical to GB:X69139, contains a gamma-thionin family signature (PDOC00725); supported by cDNA: gi_13878184_gb_AF370355.1_AF370355 | 2.35911 |
| At1g02205 | hypothetical protein contains similarity to lipid transfer protein GI:498038 from (Senecio odorus) | 2.27606 |
| At3g28530 | hypothetical protein | 2.27178 |
| At2g34850 | putative UDP-galactose-4-epimerase | 2.27167 |
| At2g18050 | histone H1 ;supported by full-length cDNA: Ceres:112970. | 2.26828 |
| At2g47780 | unknown protein | 2.25892 |
| At5g01600 | ferritin 1 precursor ;supported by full-length cDNA: Ceres:1100. | 2.22261 |
| At1g48100 | polygalacturonase PG1, putative similar to GB:AAD46483 from [Glycine max] (Mol. Plant Microbe Interact. 12 (6), 490-498 (1999)); supported by cDNA: gi_15292728_gb_AY050798.1_ | 2.12945 |
| At1g62510 | similar to 14KD proline-rich protein DC2.15 precursor (sp\|P14009); similar to ESTs emb\|Z17709 and emb\|Z47685 similar to hybrid proline-rich protein GB:CAA59472 GI:4454097 from [Catharanthus roseus] | 2.1138 |
| At5g35480 | unknown protein | 2.02394 |

***DOWNREGULATION***

| **Transcript ID** | **Target Description** | **Fold Change** |
| --- | --- | --- |
| At1g61800 | glucose-6-phosphate/phosphate-translocator precursor, putative similar to glucose-6-phosphate/phosphate-translocator precursor GI:2997591 from [Pisum sativum]; supported by cDNA: gi_14596172_gb_AY042874.1_ | 4.54335 |
| At2g43620 | putative endochitinase | 3.28131 |
| At5g65080 | MADS box transcription factor-like protein | 2.5672 |
| At1g77070 | MADS-box protein (MADS2), putative similar to MADS-box protein (MADS2) GI:886400 from [Oryza sativa] | 2.54246 |
| At3g49920 | porin - like protein porin, Mesembryanthemum crystallinum, PIR:T12558 | 2.41689 |
| At2g02100 | protease inhibitor II identical to GB:X69139, contains a gamma-thionin family signature (PDOC00725); supported by cDNA: gi_13878184_gb_AF370355.1_AF370355 | 2.35911 |
